# Autoencoder-Based Dempster-Shafer Decision-Making Framework for Intelligent Process Monitoring: Applications to Nonlinear Chemical Processes

**DOI:** 10.1021/acsomega.6c08762

**Published:** 2026-10-23

**Authors:** Remigius Nnadozie Ewuzie, Shivaneswar Gunasekaran, Zainal Ahmad, Norazwan Md Nor

**Affiliations:** School of Chemical Engineering, 65270 Universiti Sains Malaysia, Engineering Campus Seri Ampangan, Nibong Tebal, Penang 14300, Malaysia

## Abstract

Industrial chemical processes are nonlinear, large-scale, and strongly coupled, making them susceptible to disturbances that threaten operational safety. Conventional model-based and deep learning approaches often struggle with nonlinear dynamics, limited labeled fault data, detection latency, and diagnostic uncertainty. This study presents an intelligent process monitoring framework integrating autoencoder (AE)-based feature learning using denoising autoencoder (DAE), sparse autoencoder (SAE), and variational autoencoder (VAE) models with Dempster–Shafer theory (DST) for uncertainty-aware decision fusion. The framework was evaluated on a benchmark continuous stirred-tank reactor data set containing 12 fault scenarios and normal operating conditions. Baseline accuracies of 87% (DAE), 79% (SAE), and 74% (VAE) improved to 93%, 92%, and 90%, respectively, after DST integration. Following this integration, DAE-DST reduced the false alarm rate (FAR) to 1.41%, increased the G-mean to 0.911, and shortened the mean detection delay (MDD) to 0.56 min. The proposed framework provides a robust and computationally efficient solution for reliable real-time process monitoring in nonlinear chemical processes.

## Graphical abstract

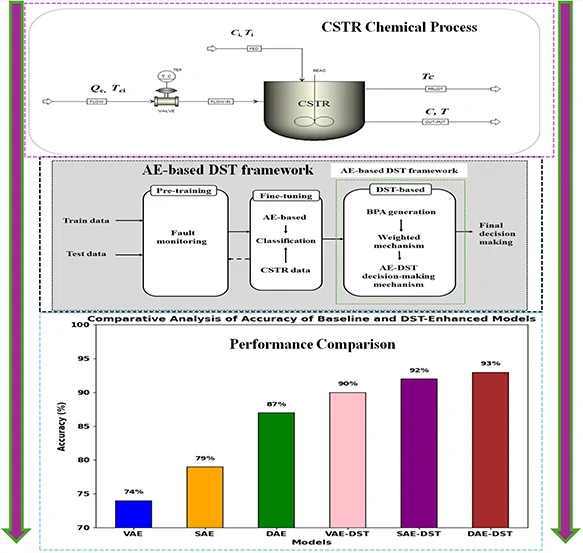

## 1 Introduction

Industrial accidents continue to occur despite extensive efforts to minimize risk and enhance safety through advanced monitoring and prevention systems.^1,2^ In chemical processes, where operations are typically large-scale, highly integrated, and nonlinear, unexpected disturbances can propagate rapidly across units through material flows and control loops, resulting in hazardous failures if not addressed promptly. Process monitoring is essential for ensuring safe and dependable operation, as it allows abnormal conditions to be detected early and their underlying causes to be identified before they develop into serious failures.^3,4^ Faults are generally defined as unpermitted deviations in system variables caused by disturbances that may ultimately trigger equipment malfunction, process instability, or even plant shutdown.^4,5^ Within process monitoring, detection refers to identifying the onset of abnormal events, while diagnosis involves classifying faults, determining their underlying causes, and tracing their propagation paths across process units.^6^

Process monitoring in chemical processes is commonly categorized into three main approaches: model-based, knowledge-based, and data-driven methods.^7–9^ Model-based monitoring uses first-principle models of process behavior, while knowledge-based monitoring relies on heuristics, expert insights, and operator experience. Both face challenges in complex plants with poorly understood reactions, nonlinear dynamics, or large-scale interconnections, and are often time-consuming to implement. These limitations have motivated the adoption of data-driven monitoring, which utilizes extensive operational data from sensors and control systems.^10,11^ Although raw process data are abundant, labeled fault data are scarce due to the rarity and irreproducibility of fault events. Consequently, supervised learning is limited, while unsupervised learning supports feature extraction and clustering without labels.^12,13^

Supervised learning enables fault classification when labeled data sets exist, while unsupervised learning extracts features and clusters data when labels are scarce. In a CSTR, data-driven monitoring can detect abnormal heat release or cooling jacket failures from multivariate sensor data, preventing unsafe operations and downtime. Traditionally, fault monitoring in chemical processes has been performed using multivariate statistical techniques such as principal component analysis (PCA),^14^ partial least-squares (PLS),^15^ and Fisher’s discriminant analysis (FDA).^4^ With the advancement of computational methods, artificial intelligence (AI) and machine learning (ML) approaches, including support vector machines (SVM)^16^ and artificial neural networks (ANN),^17^ have also been applied. These methods are valued for their simplicity, efficiency, and interpretability, making them attractive in early applications.

Chemical processes are inherently nonlinear dynamical systems due to complex reaction kinetics, transport phenomena, material and energy recycles, and strong process coupling. Nonlinear dynamics thus provides a theoretical basis for understanding system stability, dynamic responses, and fault-induced oscillatory or unstable behaviors.^18^ In strongly interconnected industrial systems, faults may trigger dynamic transitions or propagate as cascading instabilities, posing fundamental challenges to conventional fault monitoring methods that often assume quasi-linearity or stationarity. The nonlinear, dynamic, and nonstationary nature of chemical process data, combined with heavy reliance on handcrafted features and expert knowledge, limits the ability of traditional approaches to capture intrinsic nonlinear behaviors and long-range temporal dependencies.^2^ By contrast, deep learning (DL) enables automatic extraction of hierarchical nonlinear representations from raw data, thereby overcoming the limitations of manual feature engineering and shallow learning architectures.^19^ This strength enables deployment in nonlinear, complex chemical processes, including autoencoders (AEs) for anomaly detection,^20,21^ convolutional neural networks (CNNs) for fault monitoring in small modular reactors,^22^ and recurrent neural networks (RNNs) for dynamic fault diagnosis in chemical operations.^23^ Long short-term memory (LSTM) networks^24,25^ and gated recurrent units (GRUs).^26^

### 1.1 Related Work

Process monitoring is crucial for safe and efficient industrial operations,^27^ and deep learning enhances it by extracting hierarchical features, capturing nonlinear dependencies, and improving robustness under dynamic conditions. Early studies primarily explored autoencoder-based architectures for nonlinear feature extraction and robust representation learning. For example, Lu et al. developed a stacked denoising autoencoder to improve feature learning from signals affected by noise and operational fluctuations,^28^ while Chen and Li employed multiple sparse autoencoders (SAEs) for multisensor feature fusion.^29^ Deng et al. further developed a sparse stacked AE approach for fault detection and isolation.^30^ Variational architectures have also been investigated for anomaly monitoring; Zachary et al. employed a long short-term memory variational autoencoder (LSTM-VAE) for online monitoring of underwater gliders,^31^ while Qu et al. combined an echo state network with a deep autoencoder to improve diesel-engine monitoring.^32^ These studies highlight the effectiveness of AE-based and hybrid deep-learning approaches for nonlinear feature extraction and fault monitoring.

Beyond static representation learning, temporal deep-learning architectures have been increasingly adopted to characterize dynamic process behavior. Ren and Ni introduced a batch-wise LSTM encoder–decoder network for nonlinear batch-process monitoring,^33^ while Zhu et al. developed a BiGRU-based temporal learning framework for fault detection and diagnosis in complex industrial processes,^34^ Ni et al. incorporated physical constraints into a residual neural network to improve process monitoring,^35^ and Bai et al. combined CNNs with transfer learning for gas-turbine monitoring.^36^ Yang and Delpha introduced an empirical probability-density estimation approach for detecting subtle deviations obscured by noise,^37^ while Lee et al. developed a single-class deep-learning framework based on residual and recurrent structures for imbalanced fault monitoring.^38^ Chen et al. proposed a CNN–squeeze-and-excitation attention–LSTM model for the Tennessee Eastman process.^39^ Zhao et al. employed LSTM to learn dynamic features directly from raw TEP data,^40^ while Sun et al. developed a Bayesian recurrent neural network with variational dropout for improved monitoring accuracy.^41^ Chadha et al. used bidirectional LSTM to capture long-range temporal dependencies in TEP monitoring,^42^ and Han et al. developed a GRU-based framework for fault diagnosis on the TEP.^43^ These studies demonstrate the value of temporal modeling for process monitoring, but primarily emphasize predictive performance, with limited attention to uncertainty in diagnostic decisions. DL models were integrated with Dempster–Shafer theory and feature attention for TEP fault diagnosis, improving diagnostic performance and interpretability.^44^

Recent studies have increasingly incorporated spatial-temporal relationships and process topology into deep learning models. The GraphSiamese framework learns a process graph from variable interactions and uses graph convolutional networks to extract spatial-temporal slow features for industrial process quality prediction.^45^ Similarly, semisupervised multiscale graph neural networks learn scale-specific adjacency matrices and multiscale features for industrial process fault diagnosis while exploiting both labeled and unlabeled data.^46^ These approaches demonstrate a shift from conventional vector-based representations toward explicit modeling of process topology, spatial-temporal dependencies, and multiscale interactions. DAE, SAE, and VAE models, meanwhile, primarily learn nonlinear latent representations without explicitly encoding graph topology or temporal dependencies, while remaining attractive because of their relatively simple and computationally efficient architectures. Existing approaches have advanced nonlinear representation learning, temporal modeling and fault diagnosis; however, challenges remain in labeled-data scarcity, uncertainty quantification, computational efficiency, and detection latency. These gaps motivate the proposed framework for efficient and uncertainty-aware fault diagnosis.

### 1.2 Motivation

Process monitoring in chemical processes is critical for maintaining operational safety, efficiency, and reliability.^47^ Despite significant progress achieved through deep learning, several persistent limitations continue to restrict the practical deployment of existing methods. The key challenges are

#### 1.2.1 Scarcity of Labeled Fault Data

Labeled fault data are scarce, costly, and often incomplete because representative fault events are rare and require specialized expert labeling. Although supervised models such as CNNs and LSTMs can provide effective fault classification, their dependence on labeled data limits practical deployment. Semisupervised learning can mitigate this constraint by exploiting abundant unlabeled process data together with limited labeled samples, while generative approaches, including recent diffusion-based methods, offer opportunities to augment underrepresented fault conditions with process-relevant synthetic data.^48^ However, their integration with uncertainty-aware fault diagnosis remains relatively underexplored in chemical processes.

#### 1.2.2 Uncertainty Quantification in Sensor Data and Fault Classification

Industrial data are prone to noise, missing values, and overlapping fault signatures, with sensor readings affected by drift, measurement errors, and harsh environments. Many monitoring studies prioritize accuracy without addressing uncertainty, risking false alarms or missed detections in safety-critical operations. Sensor-level uncertainty arises from noise, calibration errors, and process fluctuations, while classification-level uncertainty occurs when deep learning cannot distinguish overlapping faults, especially with limited or unseen data. Ignoring these uncertainties may produce misleading alarms, reduce operator trust, and compromise real-time decisions.

#### 1.2.3 Computational Efficiency for Real-Time Monitoring

Deep architectures such as CNNs and Transformers, while powerful, often require substantial computational resources, limiting their suitability for real-time monitoring. This highlights the need for lightweight and resource-efficient solutions for online process monitoring.

#### 1.2.4 Neglect of Latency in Real-Time Monitoring

Fast response is crucial in chemical processes, as delayed fault detection can cause safety hazards or economic losses. Most studies focus on accuracy metrics while ignoring detection latency, limiting the applicability of models for time-sensitive monitoring and highlighting gaps in addressing real process conditions. Therefore, there is a pressing need for new process monitoring frameworks that

1. Reduce dependency on large labeled data sets.
2. Integrate robust mechanisms for uncertainty management.
3. Achieve computationally efficient operation suitable for online monitoring.
4. Explicitly account for detection latency in real-time fault scenarios.

Motivated by these gaps and challenges, this study proposes a new process monitoring framework.

### 1.3 Contributions

Effectively addressing data scarcity, uncertainty, computational efficiency, and latency in chemical process monitoring requires frameworks capable of learning from limited labeled data, quantifying uncertainty in sensor-based decisions, and operating efficiently in real time. In response to these challenges, this study advances the state of the art by proposing an integrated framework that combines autoencoder-based feature learning with Dempster–Shafer theory for robust and intelligent process monitoring in chemical processes. The main contributions of this work are summarized as follows:

#### 1.3.1 Development of Tailored Autoencoder-Based Models for Extracting Informative Features and Diagnosing Process Faults

This work employs three notable autoencoder variants, namely: denoising autoencoder (DAE), sparse autoencoder (SAE), and variational autoencoder (VAE), to overcome limitations associated with conventional deep learning models. DAE enhances robustness by reconstructing clean signals from noisy inputs, SAE enforces sparsity to extract compact and discriminative latent features, and VAE introduces probabilistic latent modeling to explicitly capture uncertainty in data representations. The proposed framework employs unsupervised learning and reconstruction to learn latent representations, followed by supervised fault classification, while remaining computationally lightweight compared to CNNs, LSTMs, and transformers.

#### 1.3.2 Integration of Autoencoder Outputs with Dempster–Shafer Theory for Uncertainty-Integrated Fusion

The proposed framework emphasizes uncertainty management by mapping autoencoder outputs or classification accuracies into basic probability assignments (BPAs) and fusing them using DST. This integration enables systematic representation, quantification, and redistribution of uncertainty, thereby improving diagnostic reliability under noisy, incomplete, or conflicting fault conditions.

#### 1.3.3 Computational Efficiency for Real-Time Monitoring

The AE-based DST framework is lightweight, resource-efficient, and better suited for online monitoring. It explicitly considers computational latency, ensuring that fault detection remains timely and responsive in dynamic process environments. Performance is further assessed using the false alarm rate (FAR), geometric mean (G-mean), and mean detection delay (MDD), which together reflect classification reliability, the balance between sensitivity and specificity, and the timeliness of fault detection.

#### 1.3.4 Validation on a Benchmark Chemical Process

The framework is rigorously validated on the continuous stirred tank reactor (CSTR) benchmark, a widely recognized testbed for process monitoring. The results indicate that the AE-based DST framework surpasses baseline models with respect to classification accuracy, robustness under uncertainty, and the effectiveness of uncertainty quantification.

This study develops an integrated AE-based DST framework for uncertainty-aware process monitoring and fault diagnosis, combining DAE, SAE, and VAE with DST-based decision fusion. The framework is evaluated on a benchmark continuous stirred-tank reactor data set comprising 12 fault scenarios.

The remainder of this paper is organized as follows. Section 2 presents a concise overview of the Dempster–Shafer decision-making framework, and Section 3 describes the fundamental principles of autoencoder-based models. Section 4 details the proposed process monitoring and fault diagnosis framework and its performance evaluation, while Section 5 concludes the paper and discusses potential directions for future research.

## 2 Dempster–Shafer Decision-Making Mechanism

Dempster–Shafer theory is a mathematical framework for reasoning under uncertainty that enables the representation and fusion of incomplete or conflicting information from multiple sources.^49^ Its ability to explicitly model uncertainty has led to broad applications in risk assessment, information fusion, and decision support,^50–53^ making it particularly well suited for chemical process monitoring and fault diagnosis, where noisy or heterogeneous process data can compromise the reliability of conventional classifiers. Unlike conventional fusion methods such as weighted averaging^54^ and Bayesian fusion,^55^ Dempster–Shafer theory integrates evidence from multiple models by explicitly redistributing conflicts and quantifying uncertainty through belief measures. This capability is particularly valuable when fusing heterogeneous latent representations learned by different autoencoder variants, which may yield partially conflicting predictions under nonlinear, dynamic, and nonstationary process conditions. The fundamental principles of Dempster–Shafer theory can be summarized as follows:

### 2.1 Collection of Distinct Fault Hypotheses

Let Θ = {{*F*_1_, *F*_2_, ..., *F*_*N*_}} represent a finite set of mutually exclusive fault hypotheses. This collection is referred to as the frame of discernment (FOD). The power set of Θ, denoted as 2^Θ^, contains all possible subsets of Θ, including individual faults, combinations of faults, and the empty set ϕ. For example:

1 $$2^{\Theta }=\left\{\phi ,\{F_{1}\right\},\left\{F_{2}\right\},...,\left\{F_{1},F_{2}\right\},\left\{F_{1},F_{3}\right\},....,\Theta \}$$

In chemical process monitoring, each element *F*_i_ may correspond to a specific fault type, such as a sensor bias, valve sticking, or flow disturbance.

### 2.2 Basic Probability Assignment (BPA)

The basic probability assignment (*m*) distributes belief across subsets of the frame of discernment. For any subset A ⊆ Θ, m­(*A*) ∈ [0, 1] represents the degree of belief committed exactly to A, with the properties:

2 m(ϕ)=0

3 $$\underset{A\subseteq \Theta }{\sum }m\left(A\right)=1$$

For example, in fault monitoring, a sensor may assign:

4 $$\begin{array}{l} m\left(\left\{F_{1}\right\}\right)=0.7\left(70\%\mathrm{belief that}F_{1}\mathrm{occurred}\right),\\ m\left(\left\{F_{2}\right\}\right)=0.2\left(20\%\mathrm{belief that}F_{2}\mathrm{occurred}\right)\\ m\left(\left\{F_{1},F_{2}\right\}\right)=0.1\left(10\%beliefthefaultiseitherF_{1}orF_{2}\right) \end{array}$$

This flexibility allows uncertainty to be shared between single faults and fault combinations when sensor evidence is inconclusive.

### 2.3 Belief Function (*Bel*)

The belief function (Bel) quantifies the total support that evidence provides to a subset *A* of Θ. It is defined as the sum of all BPA values of subsets *B* contained within *A*.

5 $$Bel\left(A\right)=\underset{B\subseteq A}{\sum }m\left(B\right),A\subseteq \Theta$$

In fault monitoring, if *A* = {*F*_1_, *F*_2_}, then Bel­(*A*) measures the confidence that either *F*_1_ or *F*_2_ has occurred, based on all evidence strictly supporting these faults.

### 2.4 Plausibility Function (*Pl*)

The plausibility function (*Pl*) measures how plausible a subset *A* is, considering the evidence that does not contradict it:

6 Pl(A)=1−Bel(A̅),A⊆Θ

In other words, it reflects the maximum potential support that could be assigned to *A*. For instance, in a chemical process, *Pl*({*F*_1_}) indicates how possible it is that fault *F*_1_ occurred, given all available but uncertain sensor evidence. Together, BPA, Bel, and Pl provide a structured way to represent both certainty and uncertainty in diagnosing chemical process faults, helping reduce false alarms and improving decision reliability.

### 2.5 Dempster’s Rule of Combination

During evidence fusion, Dempster’s rule provides a mechanism for combining BPAs from multiple evidence sources, with the conflict factor (K) representing the degree of disagreement between conflicting evidence. The normalization term (1-K) normalizes the combined mass assigned to the nonconflicting hypotheses. Basic DST notations are summarized in Table S1.

## 3 Autoencoder-Based Models

Autoencoders are unsupervised neural networks that learn compact latent representations by minimizing reconstruction error between inputs and their reconstructions.^56,57^ Their effectiveness in feature learning and dimensionality reduction makes them well suited for fault detection and diagnosis in settings where labeled fault data are limited.^58^ Given the input data (*x*), the latent variable can be mathematically represented as

7 $$h=f\left(W_{E}x+b_{E}\right)$$

where *f* represents the nonlinear activation function, and (*W*_E_, *b*_E_) denote the encoder parameters. The decoder maps the latent variable (*h*) back to the input space and produces the reconstructed output *x̂* in order to capture the key features.

8 $$\mathrm{x̂}=f\left(W_{D}h+b_{D}\right)$$

where (*W*_D_, *b*_D_) represent the decoder parameters. The autoencoder loss function is defined as the reconstruction error between the original input *x* and its reconstructed output *x̂*, given by

9 $$L_{AE}=\left|\right|x-\mathrm{x̂}{||}_{2}^{2}$$

The encoder–decoder parameters are optimized via backpropagation, and the resulting latent variables serve as compact feature representations of the input data.

### 3.1 Denoising Autoencoder

The denoising autoencoder enhances representation robustness by training on corrupted inputs to reconstruct clean signals, thereby preventing trivial identity mappings and improving noise tolerance.^59^ Unlike standard autoencoders, DAEs are trained on corrupted inputs to reconstruct the original uncorrupted signals (Figure 1). This training strategy enables the extraction of discriminative features and makes the model well-suited for noisy industrial chemical processes affected by disturbances or measurement errors. Mathematically, let the clean input be denoted as *x* and its corrupted version as *x̃* obtained by applying a stochastic corruption process. The corrupted input *x̃* is transformed into the latent representation h.

10 $$h=f\left(W_{E}\mathrm{x̃}+b_{E}\right)$$

where (*W*_E_, *b*_E_) are the encoder’s weights and bias parameters.

**Figure 1:**
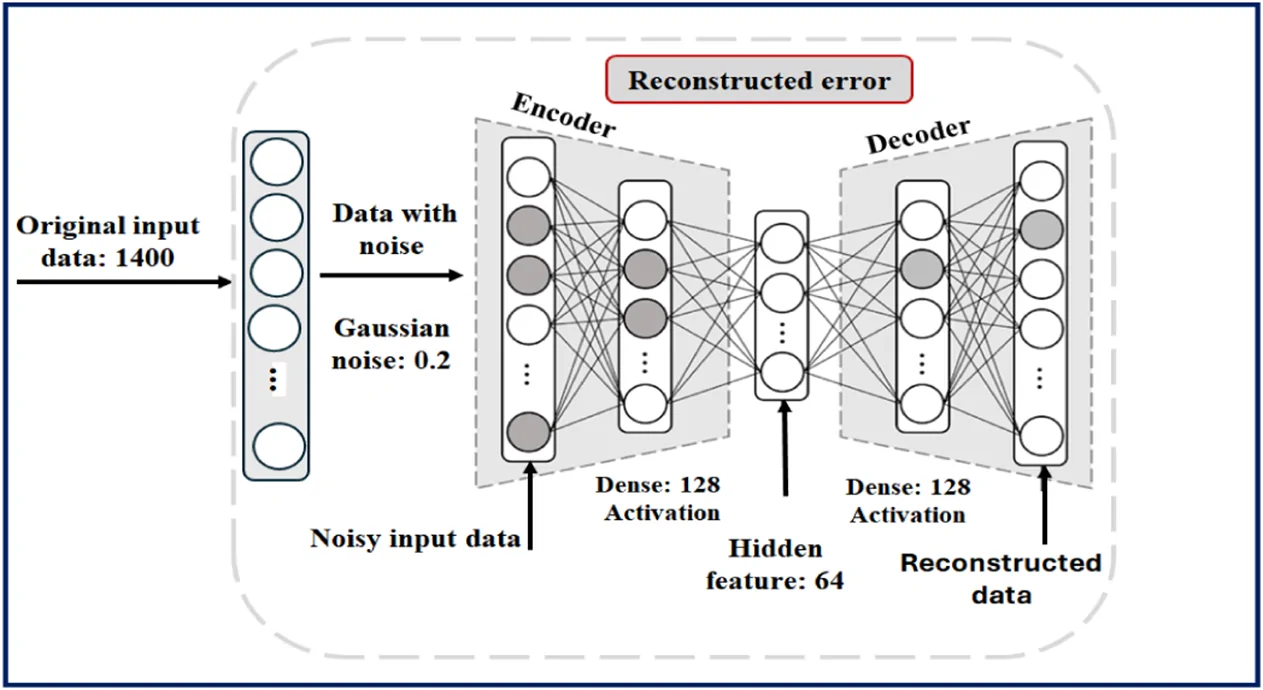
Basic architecture of a denoising autoencoder.

*f* is a nonlinear activation function.

The decoder reconstructs the clean input *x*, producing the reconstructed output *x̂*:

11 $$\mathrm{x̂}=f\left(W_{D}h+b_{D}\right)$$

with (*W*_D_, *b*_D_) denoting the decoder parameters. The loss function measures the reconstruction error between the original clean input and the decoder output:

12 $$L_{DAE}\left(x,\mathrm{x̂}\right)=\left|\right|x-\mathrm{x̂}|{|}_{2}^{2}$$

Through this denoising objective, the DAE suppresses stochastic noise while preserving essential input features.

### 3.2 Sparse Autoencoder

The sparse autoencoder enforces hidden-layer sparsity to learn compact and discriminative representations by suppressing irrelevant information and emphasizing fault-relevant features, which is particularly effective for high-dimensional and noisy process data. Sparsity is quantified by the average activation of each hidden neuron across the training samples, given by

13 pJ^=1n∑i=1naj(xi)

where, pJ^ denotes the average activation of hidden neuron *j* across all training samples, *n* is the total number of training samples, and, *a*_*j*_(*x*_*i*_) represents the activation of neuron *j*.

Sparsity is enforced by adding a Kullback–Leibler (KL) divergence penalty that drives the average activation of each hidden neuron toward the desired sparsity level *p*. The KL divergence penalty is defined as

14 ∑j=1HKL(p||pj^)=∑j=1H(plog(ppj)+(1−p)log(1−p1−p̂j))

where *p* denotes the sparsity level, and KL(p||pj^) the quantifies the discrepancy between *p* and the average activation *p̂*_*j*_ of hidden neuron *j*. When p̂j=p,KL(p||pj^)=0 . When the difference between *p* and *p̂*_*j*_ is substantial, the KL divergence yields a larger value. Accordingly, the SAE loss function can be expressed as follows:

15 LSAE(W,b)=∑i=1n||xi−xi^||22+β∑j=1HKL(p||pj^)

*H* represents the total number of neurons in the hidden layer.

KL(p||pj^) = Kullback–Leibler divergence, measuring the difference between the sparsity level *p* and the actual average activation *p̂*_j_.

*β* = the sparsity regularization parameter.

The sparsity constraint enables the SAE to capture fault-relevant features while reducing noise and overfitting.^60,61^ As illustrated in Figure 2, the encoder maps process variables into a sparse latent space, where reconstruction deviations indicate abnormal conditions.

**Figure 2:**
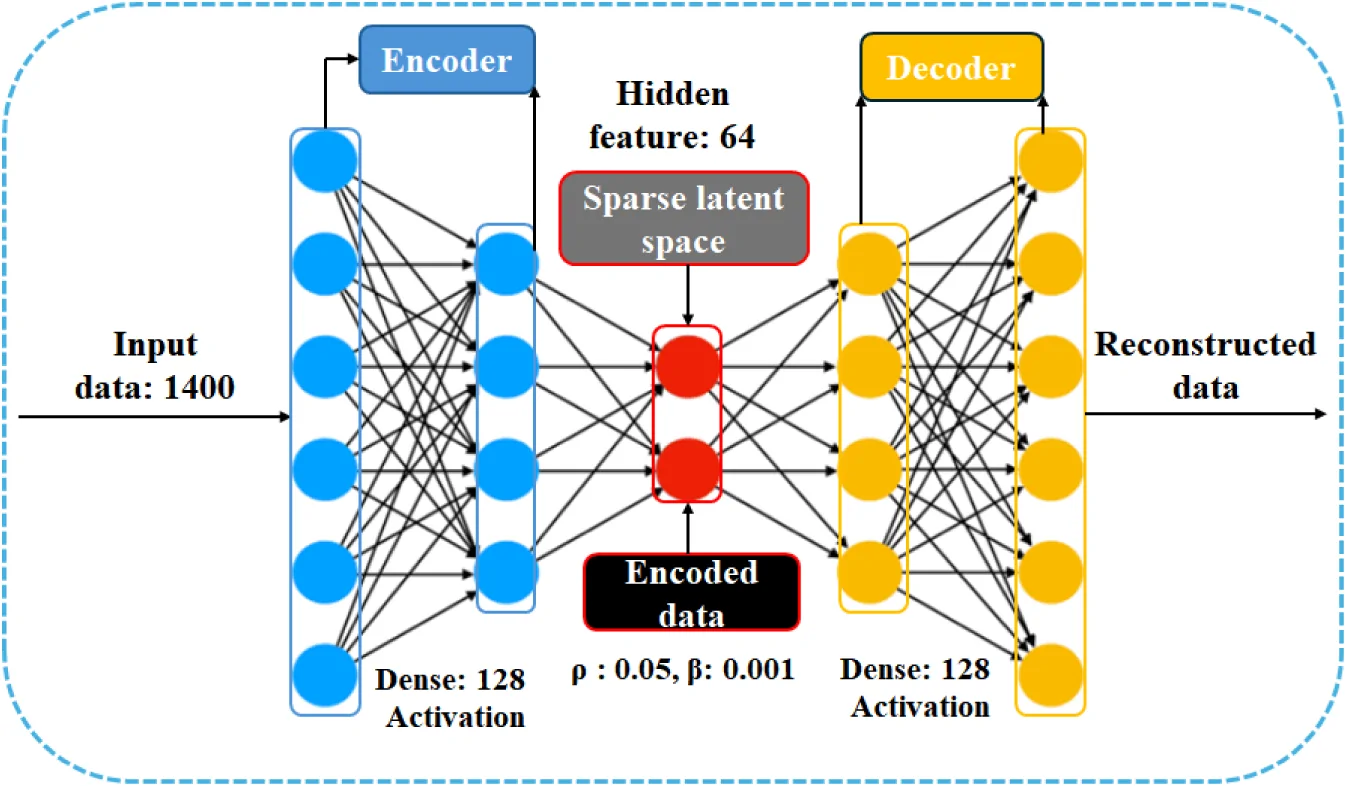
Basic architecture of a sparse autoencoder.

### 3.3 Variational Autoencoder

The variational autoencoder (VAE) extends standard autoencoders by incorporating probabilistic inference, modeling latent variables as a multivariate Gaussian to capture process variability and improve robustness,^62,63^ as illustrated in Figure 3.

**Figure 3:**
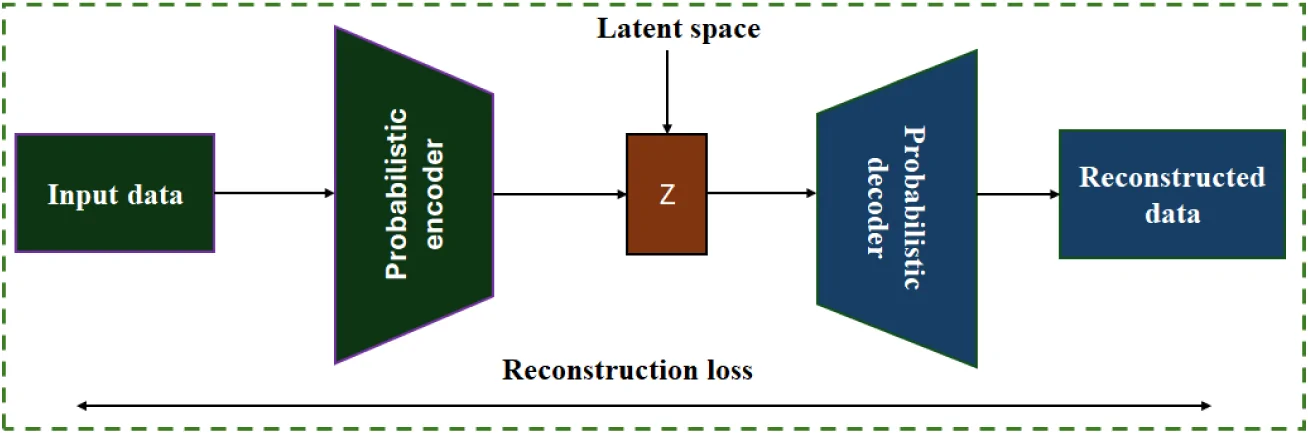
Schematic representation of a variational autoencoder.

The marginal log-likelihood is given by

16 $$\log p_{\theta }\left(x\right)=D_{KL}\left\{q_{\phi }(z|x)\left|\right|p_{\theta }\left(z|x\right)\right\}+L_{VAE}$$

While the posterior can be expressed as

17 $$p_{\theta }\left(z|x\right)=\frac{p_{\theta }\left(x|z\right)p\left(z\right)}{p_{\theta }\left(x\right)}$$

where *D*_*KL*_{*q*_*ϕ*_(*z*|*x*)||*p_θ_* (*z*|*x*)} ensures the consistency between *q*_ϕ_(*z*|*x*) and *p*_*θ*_(*z*|*x*), and is always non-negative. *L*_*V* *AE*_ represents the evidence lower bound, defined as

18 $$L_{VAE}=E_{q_{\phi }\left(z|x\right)}\left\{\log p_{\theta }\left(x|z\right)\right\}-D_{KL}\left\{q_{\phi }(z|x)\left|\right|p\left(z\right)\right\}$$

VAE treats *q*_*ϕ*_(*z*|*x*) as the encoder while *p*_*θ*_(*z*|*x*) as the true posterior.

where *D*_*KL*_{*q*_ϕ_(*z*|*x*)||*p*_*θ*_(*z*|*x*)} represents the Kullback–Leibler divergence between the approximate posterior and the prior distribution. The reconstruction term represents the expected log-likelihood of the reconstructed output and can be interpreted as the negative reconstruction loss. During VAE training, the parameters *θ* and *ϕ* are iteratively optimized. Definitions of the basic notations used in autoencoder-based models are provided in Table S2.

where, *x* = observed data (input).

*z* = latent variable.

*p*_*θ*_(*z*|*x*) = decoder network.

*q*_*ϕ*_(*z*|*x*) = encoder network.

*L*_*V AE*_ = evidence lower bound (ELBO), optimized during training.

*θ* = decoder parameters.

*ϕ* = encoder parameters.

## 4 The Proposed Integrated Process Monitoring Framework

The main contribution of this work is the development of an integrated process monitoring framework for chemical processes, demonstrated on a continuous stirred tank reactor (CSTR), which combines autoencoder-based feature learning with DST for uncertainty-aware decision fusion. By incorporating denoising, sparse, and variational autoencoders, the framework exploits complementary latent representations to characterize process dynamics under noisy, sparse, and uncertain operating conditions. The proposed approach specifically addresses the challenges inherent in multisensor and high-dimensional process environments, where heterogeneous information sources may yield conflicting diagnostic outcomes. DST is employed as a belief-based decision fusion mechanism to aggregate evidence derived from multiple latent feature representations, explicitly model uncertainty, and reconcile conflicting information. The contribution of this study, therefore, lies in systematically analyzing the interaction between uncertainty-aware DST fusion and latent features learned by different autoencoder variants and in quantitatively demonstrating the advantages of belief-based decision fusion over conventional purely probabilistic classification in chemical process fault diagnosis.

The novelty of the proposed framework lies in exploiting the complementary capabilities of different AE-based models. DAE provides noise-robust latent features, enabling reliable monitoring under imperfect or corrupted data conditions. SAE enforces sparsity in the latent space, yielding compact and discriminative features while maintaining reconstruction performance. VAE introduces probabilistic latent representations that capture underlying process variations, supporting fault monitoring under uncertainty. These complementary characteristics motivated the selection of DAE, SAE, and VAE for the proposed framework. Their outputs are subsequently fused through DST to exploit their complementary diagnostic evidence, thereby improving fault-monitoring performance and uncertainty quantification.

Figure 4 shows the proposed AE-based DST framework. Fault classification is first performed using AE models for robust feature representation with noisy or incomplete data. The resulting accuracies are then fused in the DST module to support final decisions. The AE-based–DST framework combines autoencoder-based fault classification with DST-driven decision-making for CSTR monitoring. The framework compresses high-dimensional process data into latent features, preserving fault dynamics and reducing computational load for scalable classification. The DST module fuses probabilistic outputs from the models, mitigating conflicts and reducing diagnostic uncertainty for robust online fault detection.

**Figure 4:**
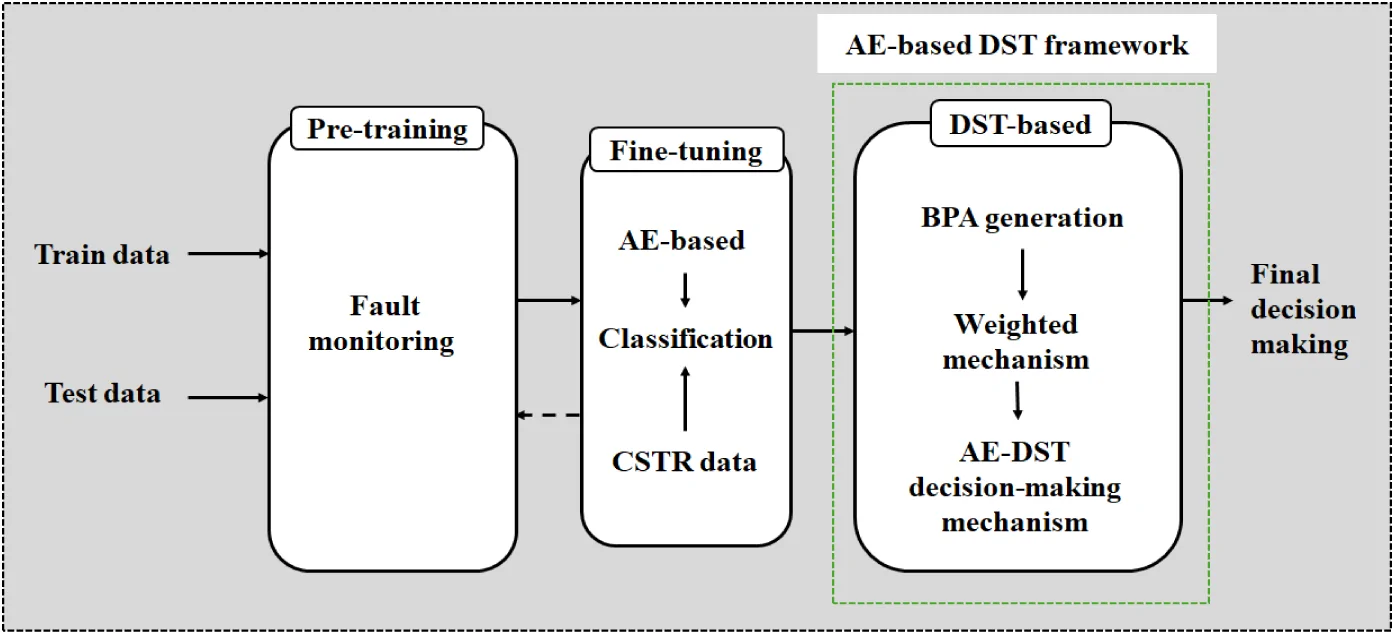
A schematic of the proposed AE-based DST framework.

### 4.1 Development of Autoencoder-Based Models for Process Monitoring

Process monitoring in CSTRs is challenging due to nonlinear dynamics, strong variable interactions, diverse fault conditions, and high-dimensional, noisy sensor data with limited fault labels. AE-based models learn compact latent representations and reconstruction features that capture fault-sensitive characteristics, enabling multisensor, multiclass fault detection and diagnosis under uncertain operating conditions. Each simulation run consisted of 200 time-point observations from seven process variables, which were flattened into a 1,400-dimensional input vector for the AE-based model.

#### 4.1.1 Development of Denoising Autoencoder for Fault Monitoring

In this study, a DAE was trained on the CSTR process data to learn compact, low-dimensional latent representations. The encoder was subsequently combined with a downstream classifier to enable supervised classification across 12 fault classes and one normal operating condition. This integrated approach allows the model to exploit noise-robust latent features for discriminative classification while preserving strong reconstruction performance for the measured process variables. The DAE was specifically designed to be resilient to measurement noise and redundancy in multivariate process variables. Its lightweight encoder–decoder architecture incorporates denoising mechanisms to enhance stability and generalization. Inputs were standardized prior to training, and Gaussian noise with a factor of 0.20 was injected into the training inputs to encourage noise-invariant feature learning.

As summarized in Table 1, the encoder begins with a fully connected layer comprising 128 neurons with ReLU activation, enabling nonlinear transformation of the input data. A 64 neuron dense layer produces the latent representation. The decoder mirrors this structure with 64 and 128 neuron layers using ReLU activation, followed by a linear output layer. The DAE was trained using Adam with a learning rate of 0.001 for 100 epochs and a batch size of 64, minimizing the MSE. Training and validation losses were monitored to assess convergence and reconstruction quality.

**Table 1:** Denoising Autoencoder Model Parameters

Parameter	Value	Description
Hidden layer 1 (encoder)	128	First dense layer of the encoder for nonlinear feature extraction
Latent dimension	64	Compressed latent feature representation
Hidden layer 1 (decoder)	64	The first decoder layer mirrors the encoder latent layer
Hidden layer 2 (decoder)	128	The second decoder layer mirrors the encoder input layer
Output layer	Linear	Reconstructs continuous-valued process variables
Noise factor	0.20	Gaussian noise was added to inputs during model training for robustness
Loss function	MSE	Mean square error
Dropout	0.2	Dropout rate for DAE regularization
Optimizer	Adam	Optimizer used
Learning rate	0.001	Initial learning rate for DAE training
Batch size	64	Training batch size
Epochs	100	Number of training epochs

#### 4.1.2 Development of Sparse Autoencoder for Process Monitoring

A sparse autoencoder was employed on the CSTR process data to learn concise, low-dimensional latent features while enforcing sparsity constraints. The encoder was subsequently combined with a downstream classifier to enable supervised classification across 12 fault classes and one normal operating condition. This integrated approach allows the model to employ sparsity-regularized latent features for discriminative tasks while simultaneously maintaining robust reconstruction performance. As shown in Table 2, the SAE architecture employs a fully connected dense layer of 128 neurons with ReLU activation, followed by a 64 dimensional latent layer with sigmoid activation to constrain latent activations between 0 and 1, facilitating sparsity through a KL-divergence penalty (desired activation ρ = 0.05, weight β 0.001).

**Table 2:** Sparse Autoencoder Model Parameters

Parameter	Value	Description
Hidden layer 1 (encoder)	128	First dense layer of the encoder for nonlinear feature extraction
Latent dimension	64	Compressed sparse latent feature representation
Hidden layer 1 (decoder)	64	The first decoder layer mirrors the latent layer
Hidden layer 2 (decoder)	128	The second decoder layer mirrors the encoder input layer
Output layer	Linear	Reconstructs continuous-valued process variables
Sparsity target (ρ)	0.05	Desired average activation of latent units
Sparsity weight (β)	0.001	Weight of KL-divergence sparsity penalty
Loss function	MSE	Mean squared error
Dropout	0.2	Dropout rate for SAE regularization
Optimizer	Adam	Optimizer used for SAE training
Learning rate	0.001	Training learning rate for the SAE model
Batch size	64	Batch size for training samples
Epochs	100	Total training iterations

The decoder comprises 64- and 128 neuron layers with ReLU activation, followed by a linear output layer for input reconstruction. The model was trained for 100 epochs by minimizing a composite loss comprising MSE and a sparsity penalty. Latent features extracted by the SAE were used to train a classifier comprising a 64 neuron hidden layer with ReLU activation, a dropout rate of 0.2, and a linear output layer for 13 classes, enabling multiclass fault classification with improved generalization.

#### 4.1.3 Development of Variational Autoencoder for Process Monitoring

In addition to the DAE and SAE, a variational autoencoder was trained on the CSTR process data to learn concise, low-dimensional latent representations of the operating conditions. The VAE uses a probabilistic encoder that projects input features into a Gaussian latent space, capturing both the mean and variance of each latent variable. The reparameterization trick was employed by expressing the latent variable as *z* = *μ* + *σ* ⊙ *ϵ*, where *μ* and *σ* denote the mean and standard deviation produced by the encoder, respectively, and *ϵ* ∼ *N*(0, *I*) is a randomly sampled noise vector. This formulation enables gradients to propagate through the stochastic sampling operation during optimization. The decoder reconstructs the input from sampled latent vectors, ensuring that the latent space encodes meaningful process information while maintaining reconstruction accuracy. To extend the VAE to supervised classification, the latent representations were subsequently combined with a downstream classifier, enabling discriminative learning across 12 fault classes and one normal operating condition (Table 3).

**Table 3:** Variational Autoencoder Model Parameters

Parameter	Value	Description
Latent dimension	32	Dimensionality of the latent space
Hidden dimension	128	Number of neurons in encoder/decoder hidden layers
KL weight	1.0	Weight assigned to the KL-divergence regularization term
Loss function	MSE + KL divergence	Combined reconstruction (MSE) and KL-divergence loss
Dropout	0.2	Dropout rate used for VAE regularization
Optimizer	Adam	Optimizer used for VAE training
Learning rate	0.001	Initial learning rate for VAE training
Batch size	64	Number of samples per training batch
Epochs	100	Number of training iterations

This hybrid approach uses the VAE’s structured latent space for fault discrimination while preserving robust reconstruction (Figure 5).

**Figure 5:**
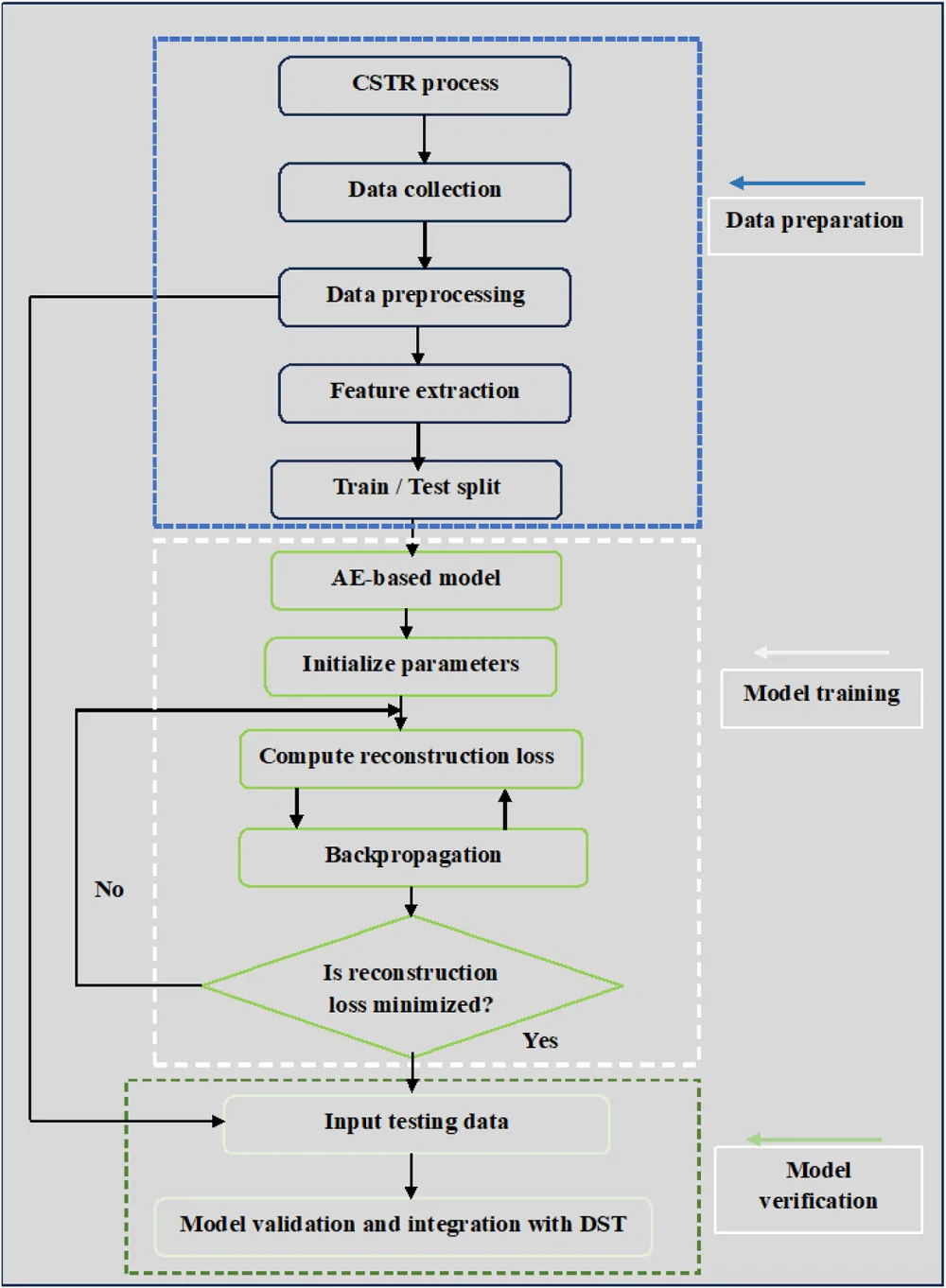
Autoencoder-based model training.

The encoder has a 128 neuron ReLU layer, followed by two linear layers producing the mean and log-variance of the 32 dimensional latent space. Latent vectors are sampled via the reparameterization trick. The decoder mirrors the encoder with a 128 neuron ReLU layer and linear output. The VAE was trained for 100 epochs with a batch size of 64 using the Adam optimizer (learning rate 0.001) and a combined MSE and KL-divergence loss. Regularization and generalization were enhanced through KL annealing and a dropout rate of 0.2. The latent dimension was evaluated at 32, 64, and 128 dimensions for each AE variant, and the configuration yielding the best diagnostic performance was selected. Accordingly, 64 latent dimensions were selected for DAE, 64 for SAE, and 32 for VAE. These values were determined empirically based on the diagnostic performance of each architecture and therefore reflect their respective representation requirements.

### 4.2 Execution Process of the Integrated Framework

Process monitoring in continuous stirred tank reactors requires continuous tracking of critical variables such as temperature, pressure, flow rates, and concentrations to ensure safe and efficient operation. Motivated by this need, the proposed AE-based DST framework is executed in five stages (**Algorithm 1**), outlining the BPA construction and fusion process, in which model outputs are converted into BPAs and combined using Dempster’s rule.

#### 4.2.1 Stage 1: Data Preprocessing

The procedure begins with importing the CSTR data set and isolating the relevant operating domain. Input process variables (*x*) are extracted and standardized to ensure uniform scaling across all measured variables. Class labels (*y*) are retained for supervised classifier training and performance evaluation, while the autoencoder is trained in an unsupervised manner.
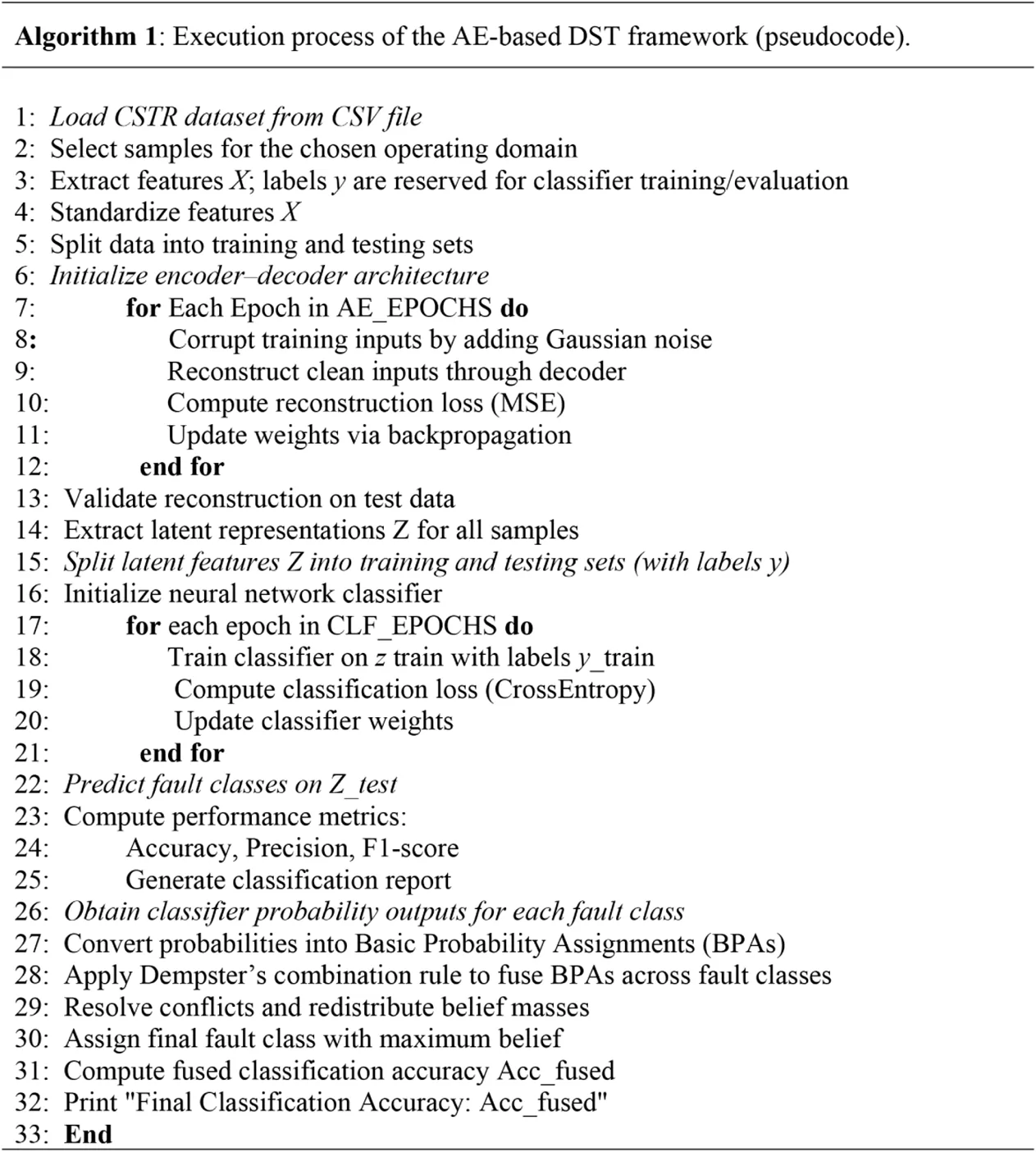


#### 4.2.2 Stage 2: Model Training

The autoencoder is designed using an encoder–decoder architecture. During training, Gaussian noise is added to the input data to enhance robustness against measurement disturbances. The model is optimized by minimizing the mean squared error (MSE) between the reconstructed outputs and the corresponding clean inputs. This training strategy encourages the encoder to learn compact, noise-invariant latent representations (*z*) that capture the underlying process dynamics. Once training converges, the encoder is retained and used to extract latent features for downstream fault classification.

#### 4.2.3 Stage 3: Classifier Training on Latent Features

The learned latent representations (*z*) are subsequently used as inputs to a supervised neural network classifier. This classifier is trained using labeled samples (*y*) to discriminate between normal and faulty operating conditions of the CSTR process. The classification objective is optimized using cross-entropy loss, ensuring that the extracted latent features are effectively mapped to discrete fault classes. The overall configuration of the proposed AE-based DST framework is presented in Figure 6. The same procedure was independently applied to each AE-based model.

**Figure 6:**
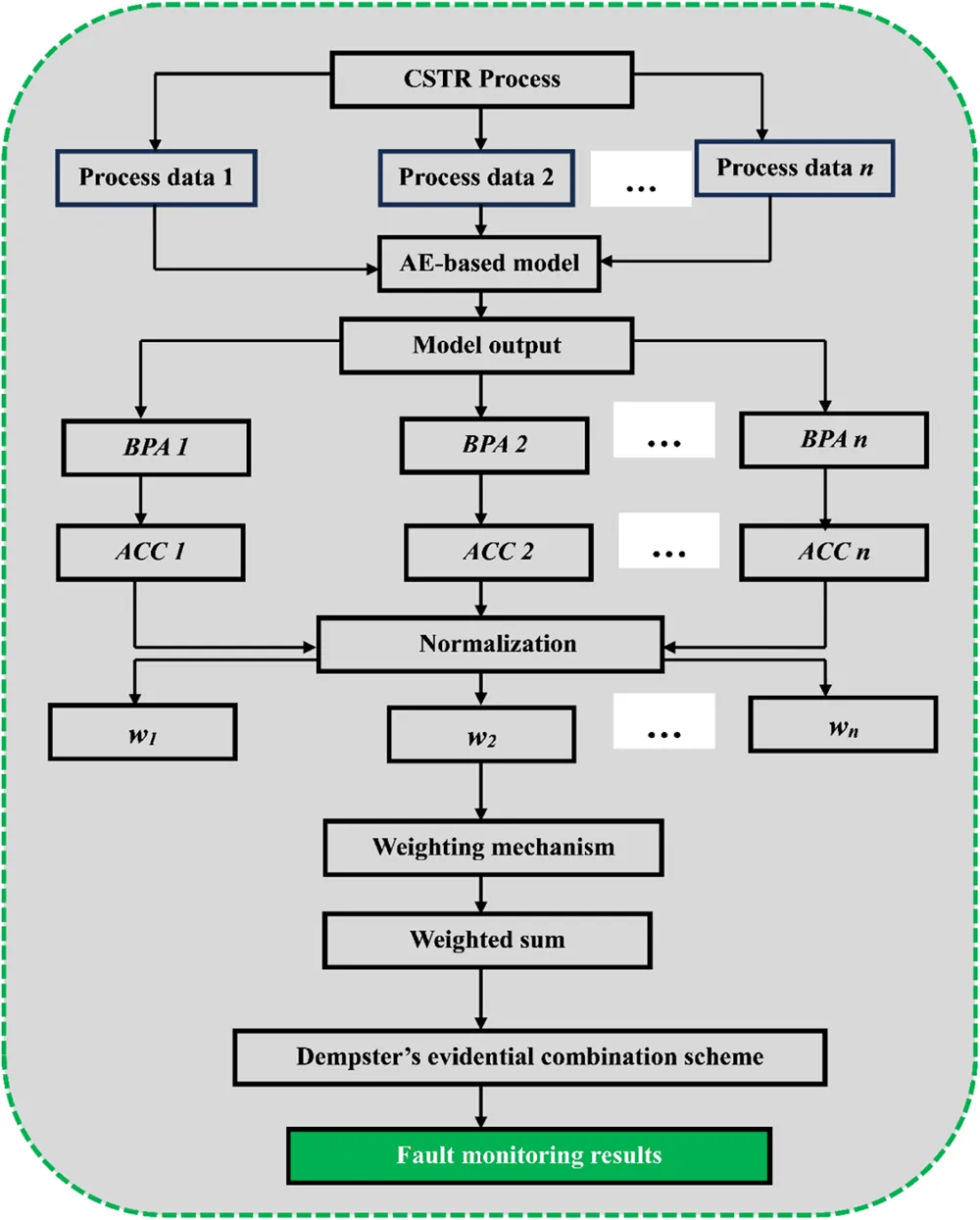
Complete framework of the proposed AE-based DST model.

#### 4.2.4 Stage 4: Model Evaluation

After training, the classifier predicts fault classes from the latent features extracted from unseen test data. Standard performance metrics, such as classification accuracy, are employed to evaluate diagnostic effectiveness. Strong classification results indicate both the quality of the learned latent representations and the reliability of the classification stage in distinguishing normal and faulty operating states.

#### 4.2.5 Stage 5: DST-Based Fusion

Rather than relying solely on raw probabilistic classifier outputs, DST is employed to explicitly model decision uncertainty in fault diagnosis. The classifier Softmax outputs are transformed into BPAs, which quantify belief mass associated with each fault hypothesis as well as the corresponding uncertainty. These BPAs, derived from multiple latent feature representations or decision instances, are then fused using Dempster’s evidential combination rule.

This iterative fusion process refines the overall diagnostic evidence by mitigating conflicts among heterogeneous diagnostic evidence sources and redistributing uncertainty across competing fault hypotheses. The final fault decision is obtained by selecting the CSTR operating state or fault class associated with the highest belief mass, and the fused classification accuracy is subsequently reported.

### 4.3 CSTR-Based Experimental Results and Analysis

In this study, a continuous flow stirred tank reactor system^14,64^ is utilized as a representative case study to validate the proposed fault monitoring framework. The CSTR, a widely recognized benchmark in chemical and environmental engineering, provides an effective platform for simulating early stage faults owing to its nonlinear and dynamic behavior.^65^ Its operation is defined by key process variables that capture the dynamic behavior of a continuously agitated reactor under steady inflow and outflow conditions, ensuring that a desired output is achieved. The reactor considered here performs an irreversible, exothermic reaction, where the feed component (*A*) is converted into the product (B). As shown in Figure 7, the reactor temperature (*T*) is regulated by adjusting the coolant flow rate (*Q*_c_), while the coolant temperature (*T*_c_) is maintained at a fixed value. An increase in reactor temperature results in a decrease in the outlet concentration (*C*), as higher feed temperatures enhance the reaction rate and consequently promote greater product formation. The CSTR process exhibits dynamic behavior described using three ordinary differential equations that represent the system’s mass and energy balances.

19 $$\frac{dC}{dt}=\frac{Q}{V}\left(C_{i}-C\right)-akC^{N}+v_{1}$$

20 $$\frac{dT}{dt}=\frac{Q}{V}\left(T_{i}-T\right)-a\frac{\Delta H_{r}kC^{N}}{\rho C_{p}}-b\frac{UA}{pC_{p}V}\left(T-T_{c}\right)+v_{2}$$

21 $$\frac{dT_{c}}{dt}=\frac{Q_{c}}{V_{c}}\left(T_{ci}-T_{c}\right)+b\frac{UA}{p_{c}C_{pc}V_{c}}\left(T-T_{c}\right)+v_{3}$$

**Figure 7:**
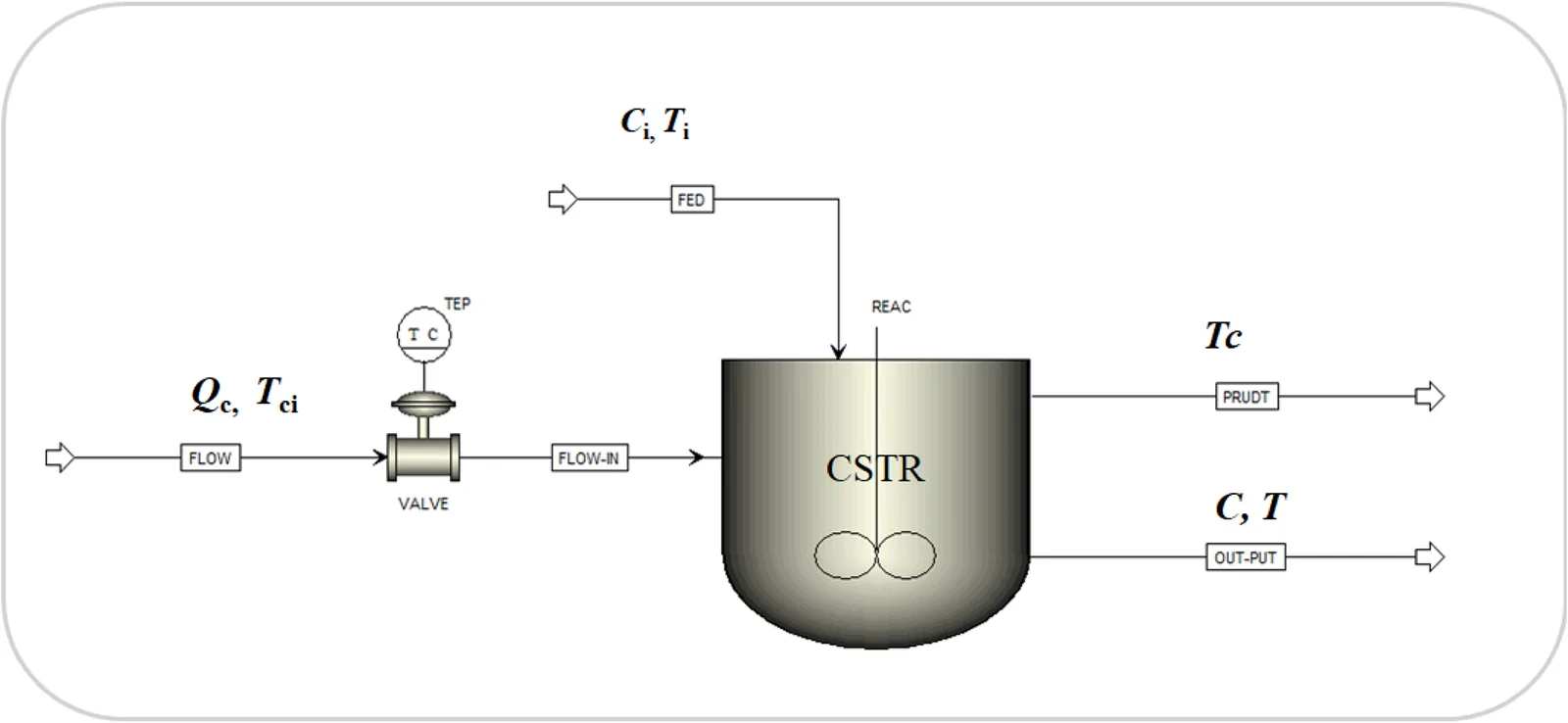
Schematic representation of the CSTR process.

The system inputs consist of the feed concentration (*C*_i_), inlet temperature (*T*_i_), and coolant inlet temperature (*T*_ci_), while the measured process variables include the coolant inlet flow rate (*Q*_c_), coolant outlet temperature (*T*_c_), reactor concentration (*C*) and reactor temperature (*T*). Process noise is denoted by *v*_*i*_, and k is the Arrhenius rate constant, given by

$$k=k_{o}\exp\left(-\frac{E}{RT}\right)$$

where, *a* = catalyst deactivation.

*b =* heat transfer fouling coefficient.

#### 4.3.1 Data Sets

The CSTR process involved seven measured variables (Table S3) and was simulated in MATLAB Simulink (R2024b), employing the ode15s solver following the methodology.^65^ The data set includes both normal and faulty operating conditions, comprising 13 classes: one normal operating class and 12 distinct fault scenarios. A total of 2,860 independent simulation runs were generated, with 220 runs assigned to each operating class, resulting in (13 × 220 = 2,860) simulation runs. For each simulation run, the seven process variables were recorded over 200 min at a sampling interval of 1 min, yielding 200 time-point observations for each variable per run. Thus, each simulation run contains (7 × 200 = 1,400) process measurements and is represented as a 1,400-dimensional feature vector. The complete data set forms a (2,860 × 1,404) matrix, where each row represents one CSTR simulation run. The first 1,400 columns correspond to process measurements, whereas the remaining four columns provide metadata on the operating class, domain identifier, process noise level, and reaction order.

Fault conditions and normal operation are encoded in column 1402, with the 12 fault scenarios listed in Table S4. The simulated faults comprise heat exchanger fouling, process disturbances, sensor bias, and catalyst deactivation. Sensor bias is modeled as a linear trend added to the measurement signals, with the noise level (ε) controlling the magnitude of variability in the data.^66^ Faults 10–12 represent random input disturbances applied to the feed concentration, reactant feed temperature, and coolant feed temperature, respectively. These disturbances are introduced at the corresponding process inputs rather than at the measurement level. Figure S1 illustrates the behavior of the concentration variable (*C*_i_) under normal operation, sensor bias, and process disturbance. Normal operation exhibits minor fluctuations, whereas sensor bias causes a steady linear drift. Process disturbances produce abrupt step-like shifts, reflecting deviations in reactor performance resulting from disturbances applied at the corresponding process input. The ground-truth fault class is determined by the process variable subjected to the disturbance; therefore, different realizations of the disturbance applied to the same process input retain the same fault label.

#### 4.3.2 Standardization of Process Variables

All numerical analyses were carried out on a 64-bit Windows 10 system featuring an Intel Core i7-4770 CPU operating at 3.40 GHz, 16 GB of system memory, and an NVIDIA graphics processor supporting CUDA acceleration. Simulations were conducted in Python 3.12.7 employing standard libraries, with SSD storage utilized to facilitate efficient data handling^67^ and Pandas was employed for data preprocessing, while Scikit-learn was utilized to compute key performance evaluation metrics. Keras was used for model development and training due to its simplicity and efficiency in constructing autoencoder architectures,^68^ with GPU acceleration via the functional API for flexible handling of complex models. CSTR process data were preprocessed to remove outliers and normalized using Z-score standardization to account for variables with differing scales, preventing bias during training. The transformation standardizes each variable by setting its mean to zero and variance to one, where μ and σ are the mean and standard deviation of the feature.

22 $$x_{n}=\frac{x-\mu }{\sigma }$$

where *x* represents the feature of the raw value and *x*_*n*_ is the normalized value.

Z-score normalization centers data at zero and scales to unit variance, improving convergence, numerical stability, and ensuring each variable contributes proportionally. For AE-based models, this enhances the extraction of meaningful latent structures from process data.

### 4.4 Performance Indicators

The performance of the process monitoring framework was assessed using accuracy, F_1_-score and precision. Accuracy quantifies the proportion of correctly predicted samples, precision evaluates the consistency in detecting specific fault classes, and the F_1_-score provides a balanced measure of recall and precision. Multiclass F_1_-score is averaged across all fault classes to provide a fair and robust assessment of detection and classification performance.^69^

23 $$Accuracy=\frac{TP+TN}{TP+TN+FP+FN}\times 100$$

24 $$Precision=\frac{TP}{TP+FP}$$

25 $$F_{1}-score=\frac{\left(2\times TP\right)}{\left(2\times TP+FN+FP\right)}$$

Following standard formulations, these metrics were derived from the counts of true positives (TP), false positives (FP), and false negatives (FN).^70^ Specifically, TP refers to the number of correctly predicted samples, FP denotes samples incorrectly classified as belonging to a class, and FN represents samples incorrectly excluded from the true class. As the F_1_-score was originally designed for binary classification, in this study, it was adapted to the multiclass setting by averaging the F_1_-scores across all fault categories.^2^ In addition to the standard classification metrics (accuracy, precision, and F_1_-score), false alarm rate (FAR), geometric mean (G-mean), and mean detection delay (MDD) were also employed to assess diagnostic performance. Inference time (ms/sample) was used as a computational efficiency metric, reflecting the latency of generating predictions, whereas MDD (min) represents the average time required to detect a fault after its onset. These metrics capture different but complementary aspects of performance. These metrics demonstrate the AE-based DST system’s robust and consistent ability to reconstruct variables, detect anomalies, and classify faults, supporting its suitability for real-time chemical process monitoring.

### 4.5 Results and Discussion

The data set was divided into 70% training and 30% validation subsets while preserving the class distribution. The DAE, SAE, and VAE models were trained using the training subset, while the validation subset was used to evaluate their performance on unseen samples.

#### 4.5.1 Visualization of Fault State Separation in AE-Based Models

To provide an intuitive representation of the recognition performance of the AE-based models for the 12 fault states and 1 normal state, the outputs are visualized in Figure 8a–c by applying t-distributed stochastic neighbor embedding (t-SNE) to the training data. Here, 0 denotes the normal data set, while the remaining labels correspond to the different fault classes. Figure 8a shows DAE latent features distinguishing most fault classes, though overlaps at the center indicate limited discriminability for some faults. In Figure 8b, the SAE latent features show much clearer separation, with reduced overlap between clusters. The sparsity constraint extracts distinctive features, producing compact fault clusters. Figure 8c shows VAE latent features forming symmetric, continuous clusters, with slight central overlaps reflecting the trade-off between smooth generative representations and class separation.

**Figure 8:**
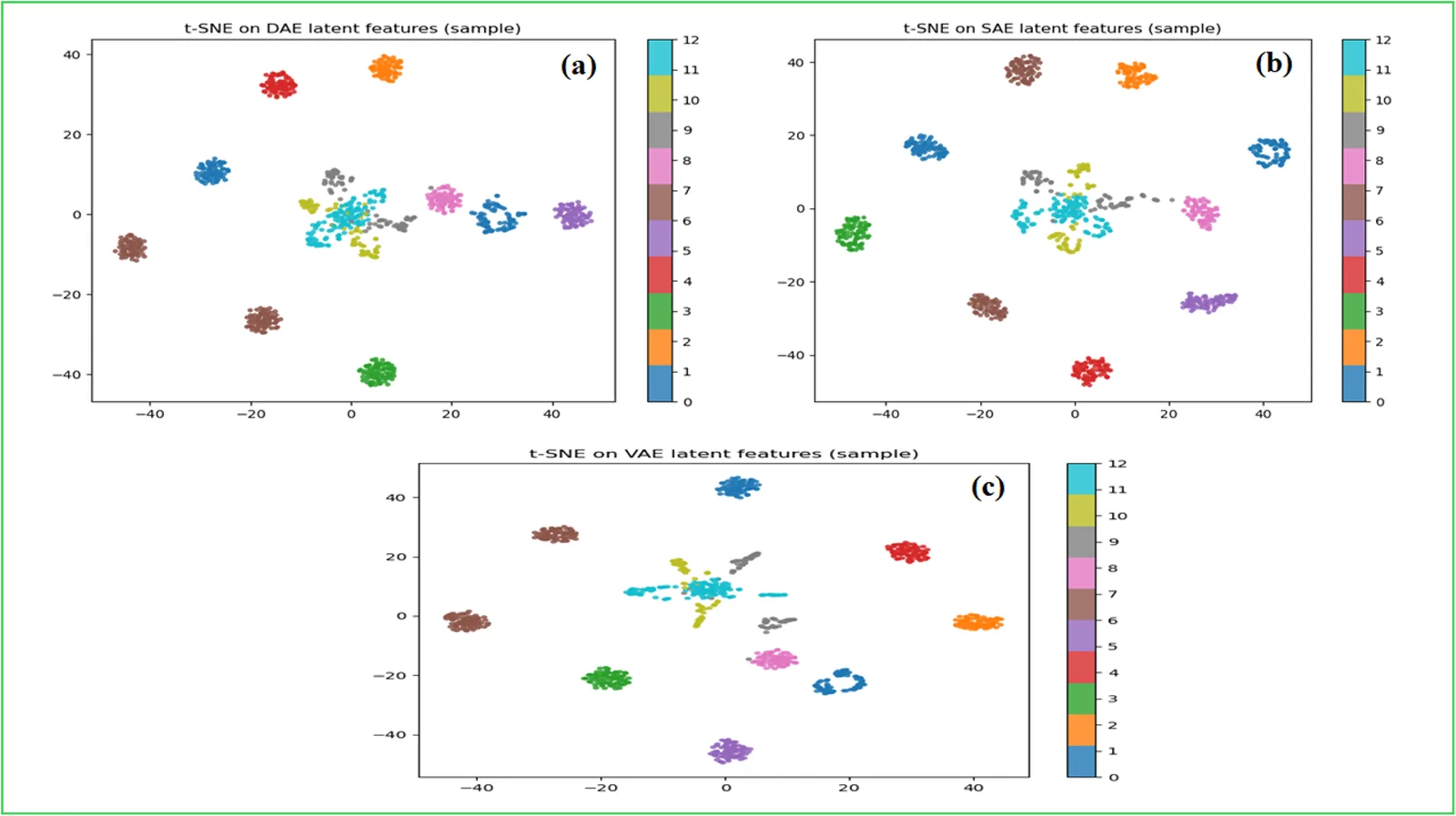
t-SNE projection of latent representations learned by AE-based models: (a) DAE, (b) SAE, and (c) VAE.

#### 4.5.2 Reconstruction Error Analysis of the AE-Based Framework

The reconstruction error analysis is used to evaluate the quality of latent representations learned by the autoencoder models rather than as a standalone fault detection statistic. The reconstruction error histograms generated by the SAE model for normal operation and the 12 fault cases are presented in Figure 9. The results show a clear and consistent separation between normal and faulty operating conditions across all fault scenarios. Normal samples exhibit reconstruction errors predominantly concentrated in the range of 1.0–1.5, with a long-tailed distribution extending to approximately 2.5, reflecting the broader variability of normal operating conditions. In contrast, faulty samples form compact distributions with low reconstruction errors (below 0.3), indicating that recurring fault patterns are reconstructed efficiently once learned by the SAE. This behavior suggests that the SAE captures simplified or more regularized dynamics associated with specific fault conditions.

**Figure 9:**
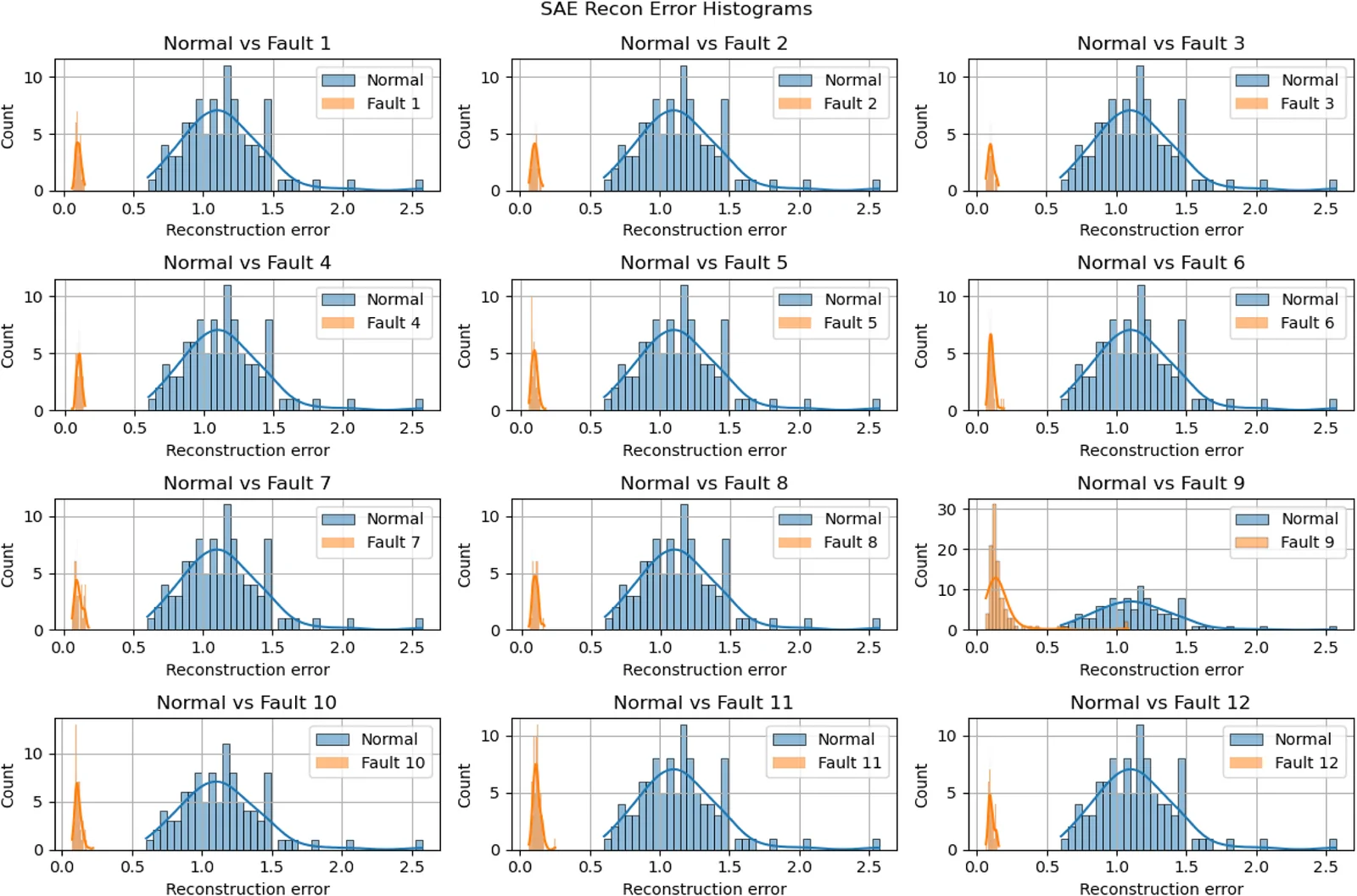
Reconstruction error analysis of a sparse autoencoder.

Although reconstruction error alone is not used as a fault detection criterion in this study, the observed separation demonstrates the discriminative capability of the learned latent representations. These representations provide informative inputs for the subsequent supervised classification stage and support reliable decision fusion when integrated with the Dempster–Shafer framework. The consistent clustering behavior across all fault cases highlights the robustness of the feature extraction process under diverse operating conditions.

#### 4.5.3 Performance Evaluation of Autoencoder-Based Models for Process Monitoring

The DAE model performance evaluation is presented in Table 4. The denoising autoencoder achieved consistently high performance across most fault categories, with perfect accuracy and precision for several faults (F2, F3, F5, F6, F7) and near-perfect results for others (F1, F4, F8). However, the model exhibited reduced performance in detecting F9, F10, F11, and F12, where accuracy dropped to 40–77%. In particular, Faults 10–12 correspond to process disturbances in feed concentration, reactant feed temperature, and coolant feed temperature, all of which were simulated as random variations. These stochastic fluctuations resemble normal process noise, making them harder to distinguish in the latent space. The DAE achieved 87% accuracy, 86% F_1_-score, and 87% precision, demonstrating strong overall diagnostic capability but difficulty with disturbance-type faults.

**Table 4:** Denoising Autoencoder Performance Metrics

Fault number	Accuracy (%)	F_1_–Score (%)	Precision (%)
Fault 1	100	97	94
Fault 2	100	100	100
Fault 3	100	100	100
Fault 4	100	98	97
Fault 5	100	100	100
Fault 6	100	100	100
Fault 7	100	100	100
Fault 8	100	98	97
Fault 9	57	59	61
Fault 10	77	67	59
Fault 11	57	57	57
Fault 12	40	49	63
Average	87	86	87

The process monitoring results for the CSTR process based on SAE are presented in Table 5. Similar to the DAE, the sparse autoencoder demonstrated excellent classification accuracy for the majority of fault categories, with perfect or near-perfect results across F1–F8, where both precision and F_1_-scores remained consistently high. However, the model’s performance degraded substantially for Faults 9–12, with accuracy dropping as low as 7% for F12 and F_1_-scores ranging only between 10–43%. These faults involve coolant flow bias and random variations in feed concentration and temperatures, which are hard to detect because subtle deviations and noise-like disturbances are difficult to isolate in the latent space.

**Table 5:** Sparse Autoencoder Performance Metrics

Fault number	Accuracy (%)	F_1_–Score (%)	Precision (%)
Fault 1	100	98	97
Fault 2	100	100	100
Fault 3	100	98	97
Fault 4	100	98	97
Fault 5	100	100	100
Fault 6	100	100	100
Fault 7	100	100	100
Fault 8	100	100	100
Fault 9	97	43	28
Fault 10	67	12	67
Fault 11	20	30	60
Fault 12	7	10	13
Average	79	75	80

The SAE’s sparsity constraint captures dominant features but reduces sensitivity to small or noise-like deviations, contributing to misclassifications. It achieved 79% accuracy, 75% F_1_-score, and 80% precision, performing well for structured faults. In addition to the DAE and SAE, a variational autoencoder was employed for fault monitoring in the CSTR process, with results summarized in Table 6.

**Table 6:** Variational Autoencoder Performance Metrics

Fault number	Accuracy (%)	F_1_–Score (%)	Precision (%)
Fault 1	100	67	51
Fault 2	100	100	100
Fault 3	100	95	91
Fault 4	100	98	97
Fault 5	100	100	100
Fault 6	100	65	48
Fault 7	100	100	100
Fault 8	7	13	100
Fault 9	60	64	69
Fault 10	40	38	35
Fault 11	60	68	78
Fault 12	13	51	34
Average	74	70	75

The VAE achieved strong diagnostic capability for several faults, with perfect accuracy for F1–F7 and particularly high precision and F_1_-scores for F2, F3, F4, F5, and F7, demonstrating its effectiveness in capturing structured and distinct anomalies. However, the model exhibited substantial performance drops for faults F8–F12. For instance, accuracy declined to 7% for F8 and 13% for F12, while F_1_-scores for these categories ranged only between 13–68%. These categories include a step-type sensor bias (F8) and random variation disturbances in feed concentration, reactant feed temperature, and coolant feed temperature (F10–F12), along with the step disturbance in coolant flow rate (F9).

Such faults either mimic normal operating fluctuations or introduce subtle deviations that are difficult to separate in the compressed latent space. The probabilistic latent representation in the VAE, while advantageous for modeling overall variability, tends to smooth out fine-grained distinctions, reducing its sensitivity to weak or noise-like fault signatures. Furthermore, the Kullback–Leibler (KL) divergence regularization promotes continuity in the latent space, which can further suppress the detection of rare or subtle anomalies. On average, the VAE achieved 74% accuracy, 70% F_1_-score, and 75% precision, reflecting reasonable diagnostic performance but highlighting its limitations in distinguishing disturbance-type and sensor-bias faults.

Table 7 presents the classification accuracies of DAE, SAE, and VAE across the 12 CSTR fault categories. The models achieve average accuracies of 87%, 79%, and 74%, respectively, with DAE exhibiting the highest overall accuracy.

**Table 7:** Fault Classification Accuracies for the 12 Classes

Faults/Models	DAE	SAE	VAE
Fault number	Accuracy (%)	Accuracy (%)	Accuracy (%)
Fault 1	100	100	100
Fault 2	100	100	100
Fault 3	100	100	100
Fault 4	100	100	100
Fault 5	100	100	100
Fault 6	100	100	100
Fault 7	100	100	100
Fault 8	100	100	7
Fault 9	57	97	60
Fault 10	77	67	40
Fault 11	57	20	60
Fault 12	40	7	13
Average	87	79	74

#### 4.5.4 Process Monitoring Performance Using ROC and Confusion Matrices

In order to gain deeper insight into the fault monitoring results, the performance of the autoencoder-based models (DAE, SAE, and VAE) was evaluated using receiver operating characteristic (ROC) curves and multiclass confusion matrices across 12 representative CSTR fault categories, providing a comprehensive assessment of their discriminative power, classification accuracy, and robustness. As shown in Figure 10, the DAE model achieved perfect discrimination for the first nine fault types (Faults 0–8), with each class attaining 100% accuracy and no evidence of cross-class misclassification. This demonstrates that the latent representations learned by the DAE are highly effective in capturing separable fault characteristics when the underlying fault signatures are distinct. In contrast, notable misclassification was observed among Faults 9–12, which exhibit overlapping latent features.

**Figure 10:**
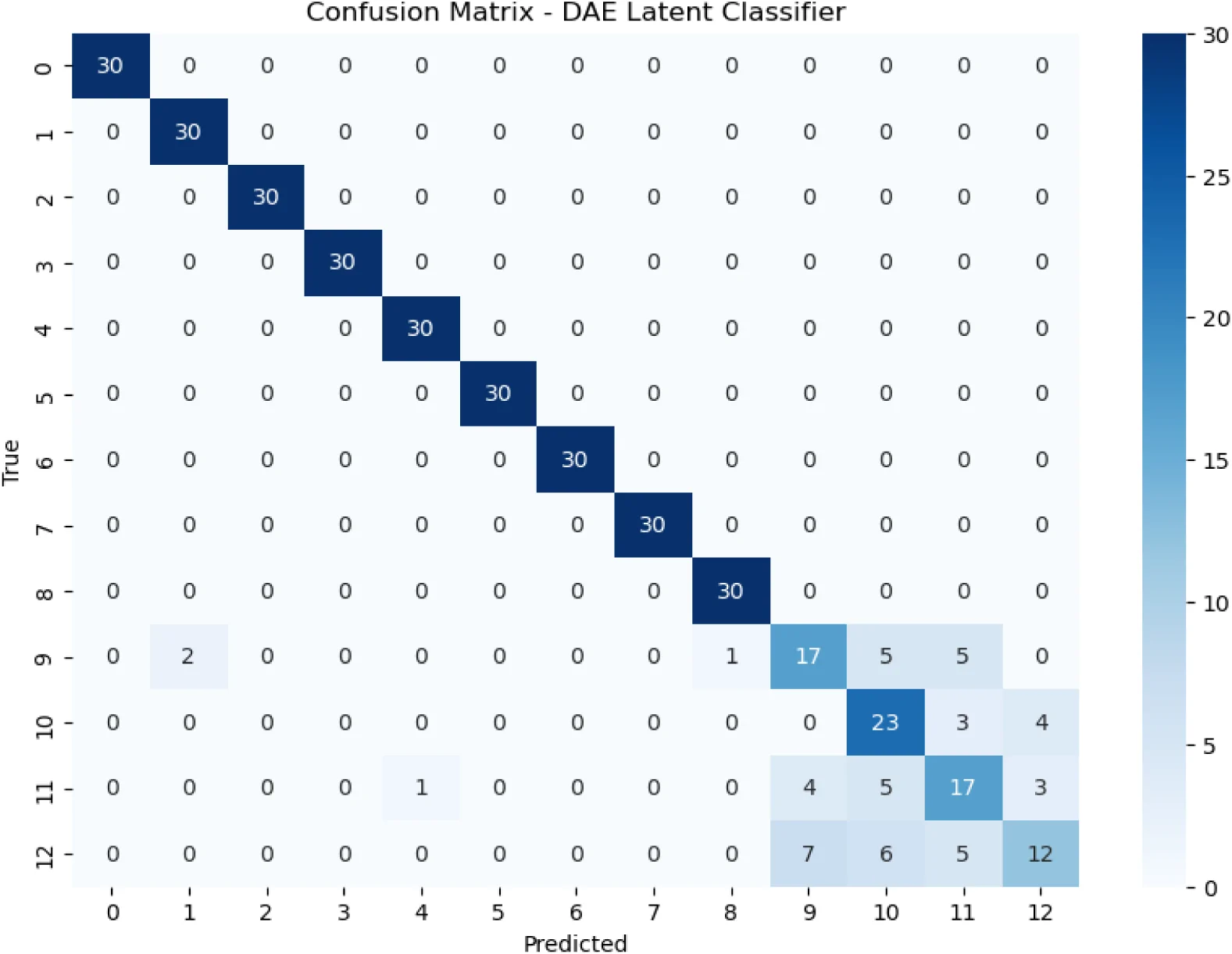
Confusion matrix of the denoising autoencoder for CSTR process monitoring.

Specifically, Fault 12, corresponding to a disturbance in coolant feed temperature, showed the poorest recognition performance, with only 40% of samples correctly identified and the remainder distributed across Faults 9–11. Likewise, Faults 9–11, representing a coolant flow rate sensor bias and process disturbances in feed concentration and reactant feed temperature, achieved moderate accuracies between 57–77%, with most errors occurring within this subgroup. The overall classification accuracy of the DAE was approximately 87.7%, highlighting both its robustness in handling linearly separable fault classes and its limitations in discriminating strongly correlated operating anomalies.

These misclassifications can be attributed to the similar dynamic responses of the process variables under these disturbances, which produce overlapping patterns in the latent feature space. In particular, thermal and flow-related deviations (Faults 9–12) tend to manifest in coupled changes in reactor temperature, concentration, and coolant dynamics, making them difficult for the latent encoder to separate. These results indicate that the DAE latent space produces compact and discriminative features for most fault types. The corresponding SAE and VAE confusion matrices are presented in Figures S2 and S3.

To further evaluate the effectiveness of the proposed DAE-based fault monitoring framework, the multiclass receiver operating characteristic analysis was conducted on the latent features extracted from the CSTR process data. This analysis demonstrates excellent classification performance, with a microaverage AUC of 0.995 and a macro-average AUC of 0.987. Classes 0–8 achieved perfect AUC scores of 1.000, indicating complete separability in latent space for faults with distinct signatures, whereas Classes 9–12 showed slightly lower AUCs (0.941–0.965), reflecting latent feature overlap and correlated disturbances such as feed and coolant variations (Figure 11). The ROC curves for Classes 0–8 rise steeply toward the top-left corner, indicative of high true positive rates with negligible false positives, while Classes 9–12 increase more gradually, revealing trade-offs between sensitivity and specificity.

**Figure 11:**
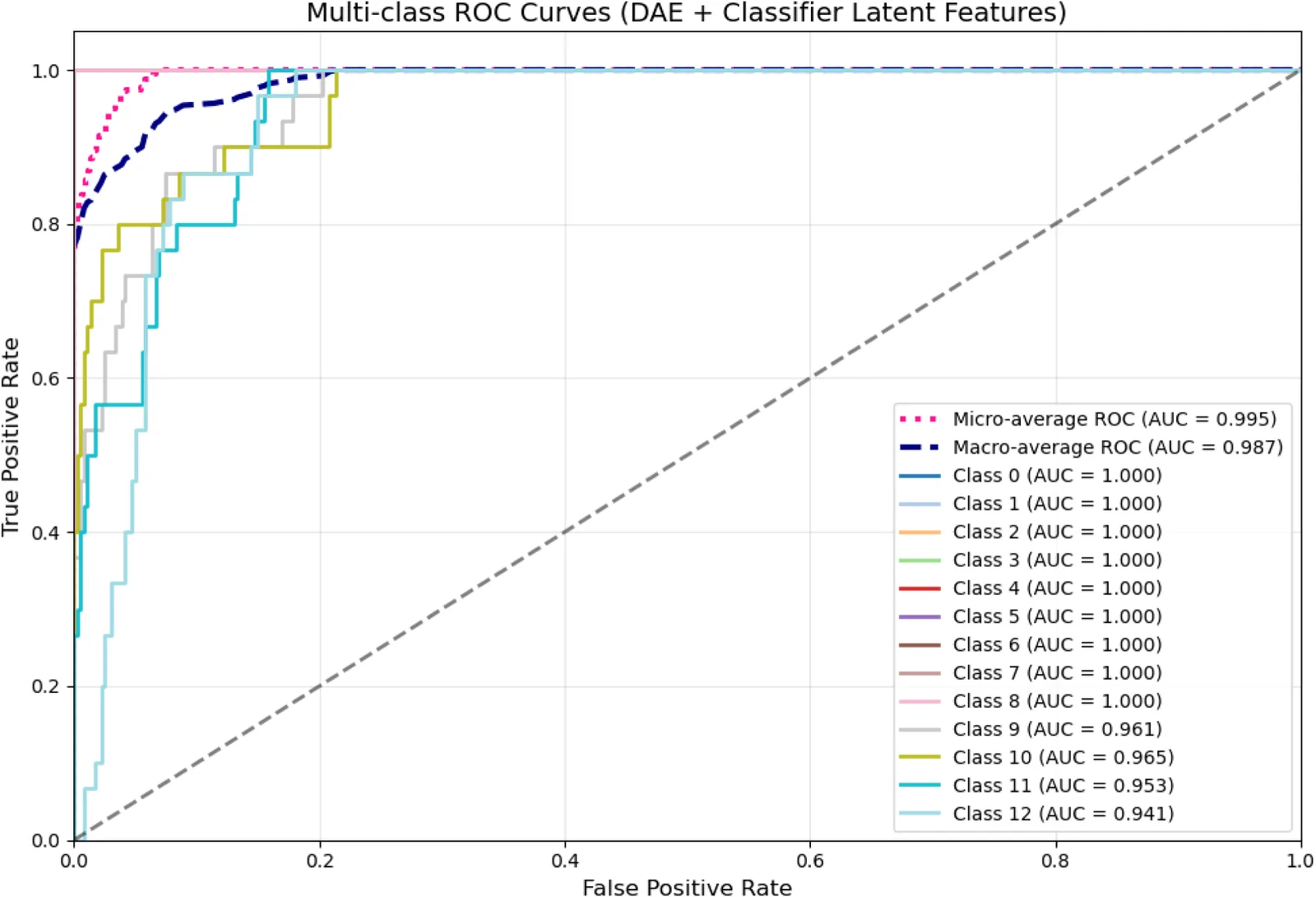
Multiclass ROC analysis of DAE-derived latent features for CSTR process monitoring.

These results confirm the DAE’s effectiveness for linearly separable faults while highlighting minor limitations for coupled or subtly distinct fault types, guiding potential avenues for further refinement in fault discrimination. The corresponding SAE and VAE ROC analyses are presented in Figures S4 and S5. Overall, the ROC and confusion matrix analyses show that the DAE model consistently achieves superior fault discrimination and classification across most fault categories. These results align closely with the average classification accuracies in Table 7, confirming the robustness and reliability of the AE-based fault monitoring framework.

The enhanced robustness of DAE can be attributed to its denoising capability, which encourages the model to learn essential, noise-invariant features and produces more discriminative latent representations.

#### 4.5.5 Performance and Computational Efficiency of the Denoising Autoencoder

As summarized in Table 8, the comprehensive analysis of the DAE model’s performance and computational efficiency highlights its significance for real-time fault detection in complex processes. High accuracy metrics, peak validation and test accuracy of 86.92%, along with an average fault detection rate of 86.92%, demonstrate effective convergence, robust feature learning, and consistent fault detection across diverse classes. Low reconstruction errors (training MSE = 0.0704, validation MSE = 0.1219) indicate a good model fit and strong generalization without overfitting.

**Table 8:** Performance and Computational Efficiency of the Denoising Autoencoder

Metric	Value	Major findings
Peak validation accuracy	86.92%	Achieved at 100 epochs
Test Accuracy	86.92%	Evaluated on 390 unseen samples
Average FDR	86.92%	Average fault detection rate across classes
Final training MSE	0.0704	Low reconstruction error
Final validation MSE	0.1219	Robust feature learning
Number of epochs run	100	Stable convergence over full training
Total training time per epoch	0.135 s	Lightweight per-epoch runtime
Total training time (100 epochs)	13.48 s	Entire training completed in under 15 s
Alternative per-epoch time (CPU-time)	0.0053 s	Excluding data-loading and I/O overhead
Inference latency (per sample)	0.0026 ms (∼384k samples/s)	Real-time suitability
Inference latency (batch mode)	0.0015 ± 0.0013 ms/sample	Faster with batching

Equally important, the model is computationally efficient: stable convergence is achieved in 100 epochs (13.5 s total training), with minimal per-epoch and per-sample computation times (0.135 s and 0.0026 ms, respectively), supporting both rapid retraining and real-time monitoring. Batch inference further reduces latency, emphasizing the model’s suitability for high-throughput, time-critical process applications.

#### 4.5.6 Performance Comparison with Baseline Autoencoder Models

The performance of the autoencoder-based fault-specific models was successfully integrated into the DST framework by using their diagnostic accuracies to derive normalized reliability weights for the 12 fault hypotheses. Specifically, the accuracy of each fault-specific model was normalized with respect to the sum of the accuracies across all 12 fault types. These normalized values quantify the relative reliability of the corresponding diagnostic models and are subsequently used to determine the mass assigned to each fault hypothesis during evidence fusion within the DST framework. The BPA represents the reliability-based mass assigned to each fault hypothesis, derived from the normalized classification accuracy of the corresponding fault-specific diagnostic model, while DST provides the mechanism for combining the resulting evidence, as defined below.

26 $$m\left(\left\{F_{i}\right\}\right)=w_{i}=\frac{ACC_{i}}{\overset{n}{\underset{j=1}{\sum }}ACC_{j}}$$

27 $$\mathrm{Weighted sum}=\overset{n}{\underset{i=1}{\sum }}w_{i}.ACC_{i}$$

The outputs of the DAE, SAE, and VAE were treated as independent diagnostic evidence sources. Table 9 summarizes the accuracies, BPAs, and weighted contributions (*ω*_1_ × *Acc*_1_) for each fault type, using the DAE as a representative example because it achieved the highest overall diagnostic performance. The same procedure was applied to the SAE and VAE outputs before DST fusion. For Faults 1–8, the DAE achieved an accuracy of 1.00, resulting in a BPA of 0.097, a weighted contribution of 0.097, and a weighted reliability contribution of 9.70% for each fault. Fault 9 (accuracy = 0.57) yielded a BPA of 0.0553, a weighted contribution of 0.0315, and a weighted reliability contribution of 3.15%.

**Table 9:** Accuracy Values, BPA, and Weighted Sum Contributions for Fault Conditions

Fault number	Accuracy	BPA	Weighted sum	Weighted reliability contribution (%)
Fault 1	1.00	*m*({*F*_1_}) = 0.097	*ω*_1_ × *Acc*_1_ = 0.097	9.70
Fault 2	1.00	*m*({*F*_2_}) = 0.097	*ω*_2_ × *Acc*_2_ = 0.097	9.70
Fault 3	1.00	*m*({*F*_3_}) = 0.097	*ω*_3_ × *Acc*_3_ = 0.097	9.70
Fault 4	1.00	*m*({*F*_4_}) = 0.097	*ω*_4_ × *Acc*_4_ = 0.097	9.70
Fault 5	1.00	*m*({*F*_5_}) = 0.097	*ω*_5_ × *Acc*_5_ = 0.097	9.70
Fault 6	1.00	*m*({*F*_6_}) = 0.097	*ω*_6_ × *Acc*_6_ = 0.097	9.70
Fault 7	1.00	*m*({*F*_7_}) = 0.097	*ω*_7_ × *Acc*_7_ = 0.097	9.70
Fault 8	1.00	*m*({*F*_8_}) = 0.097	*ω*_8_ × *Acc*_8_ = 0.097	9.70
Fault 9	0.57	*m*({*F*_9_}) = 0.0553	*ω*_9_ × *Acc*_9_ = 0.0315	3.15
Fault 10	0.77	*m*({*F*_10_}) = 0.0747	*ω*_10_ × *Acc*_10_ = 0.0575	5.75
Fault 11	0.57	*m*({*F*_11_}) = 0.0553	*ω*_11_ × *Acc*_11_ = 0.0315	3.15
Fault 12	0.40	*m*({*F*_12_}) = 0.0388	*ω*_12_ × *Acc*_12_ = 0.0155	1.55

Fault 10 (accuracy = 0.77) yielded a BPA of 0.0747, a weighted contribution of 0.0575, and a weighted reliability contribution of 5.75%. Fault 11 (accuracy = 0.57) yielded a BPA of 0.0553, a weighted contribution of 0.0315, and a weighted reliability contribution of 3.15. Fault 12 (accuracy = 0.40) yielded a BPA of 0.0388, a weighted contribution of 0.0155, and a weighted reliability contribution of 1.55%. The BPAs are normalized to satisfy the DST requirements, while the weighted contributions represent the influence of each fault class in the fusion process. Detailed step-by-step computations are provided in the (Supporting Information (eqs S1–S7) Figure S6). Fault-level analysis indicates that DST-based fusion resolves conflicting latent evidence from multiple autoencoders, improving the discrimination of overlapping faults. Faults 1–8 dominate the evidence distribution due to near-perfect accuracy, while Faults 9, 11, and 12 contribute less because of lower performance. This validates the BPA formulation and highlights the effective integration of autoencoder-based monitoring with DST for managing classification uncertainty. By quantifying belief and plausibility (Table S5), the framework enhances diagnostic robustness in the CSTR process, supporting more reliable decisions under uncertainty.

Table 10 clearly highlights the superiority of the integrated AE-based DST framework compared to standalone AE models. While the base autoencoders (VAE, SAE, DAE) demonstrate competent fault monitoring capabilities, their performance is constrained by classification uncertainty and sensitivity to overlapping fault features. By integrating these models with DST, a significant performance gain is observed across all metrics, validating the rationale behind the framework’s development. For instance, DAE alone achieves 87% accuracy, which increases to 93% with DAE–DST, accompanied by balanced precision (90%) and F_1_-score (90%). Similarly, VAE improves from 74% accuracy, 75% precision, and 70% F_1_-score to 90%, 84%, and 82%, respectively, under VAE–DST. In the case of SAE, performance rises from 79% accuracy, 80% precision, and 75% F_1_-score to 92%, 91%, and 92% with SAE–DST. These results confirm that the evidence fusion mechanism of DST not only minimizes misclassification in fault assignment but also strengthens decision reliability through the use of belief and plausibility measures. The comparative improvements validate the theoretical foundation that AE-derived latent features provide a robust basis for evidence allocation, and when embedded within the DST framework, they yield a more reliable and uncertainty-resilient fault monitoring system for complex chemical processes such as the CSTR.

**Table 10:** Comparative Performance Analysis of Baseline and DST-Enhanced Models

Models	Accuracy (%)	Precision (%)	F_1_-score (%)
VAE	74	75	70
SAE	79	80	75
DAE	87	87	86
VAE-DST	90	84	82
SAE-DST	92	91	92
DAE-DST	93	90	90

To better illustrate the comparative performance, Figure 12 presents the diagnostic accuracies of the baseline autoencoders and their DST-enhanced counterparts. The VAE model achieves an average accuracy of 74%, while VAE-DST improves to 90%, reflecting a gain of 16%. The SAE baseline records 79% accuracy, increasing to 92% with SAE-DST, a 13% improvement. Similarly, the DAE model achieves 87%, which rises to 93% with DAE-DST, corresponding to a 6% gain. In terms of overall performance, DAE-DST delivers the highest diagnostic accuracy of 93%, whereas VAE lags behind with 74%. This represents a significant performance gap of 19% (26% relative gain), highlighting the superior capability of the DAE-DST framework in capturing fault-related dynamics and reducing classification uncertainty. Importantly, this margin becomes particularly significant in the CSTR process, where overlapping fault signatures, strong nonlinearities, and sensor noise make accurate fault monitoring highly challenging.

**Figure 12:**
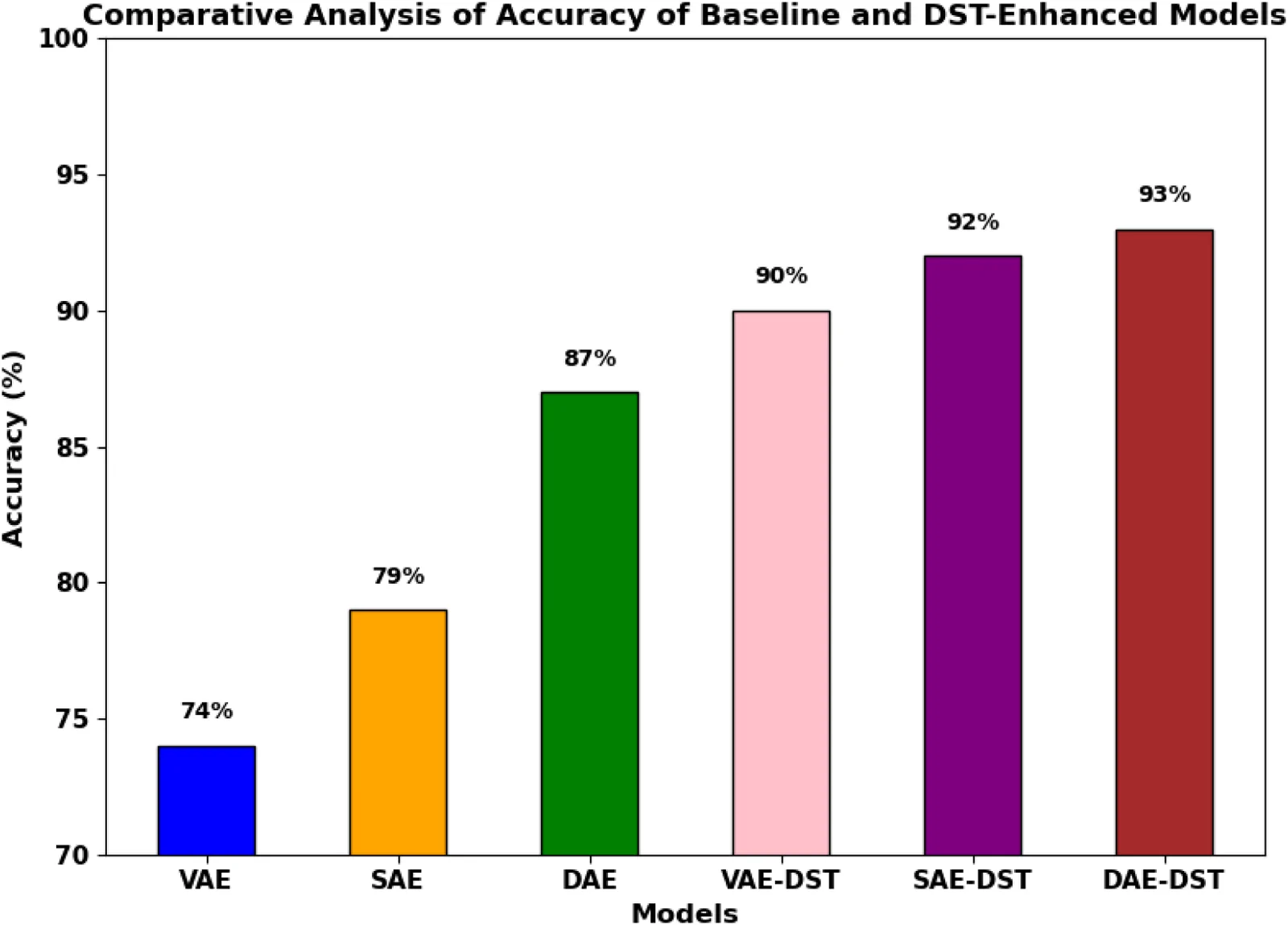
Comparative analysis of the accuracy of baseline and DST-enhanced models.

The results clearly demonstrate that while baseline autoencoders provide useful latent representations, their diagnostic effectiveness is significantly strengthened when integrated with DST, thereby establishing the AE-based DST framework as a more robust and reliable solution for intelligent fault monitoring in chemical processes.

In addition to evaluating overall accuracy, it is essential to examine how diagnostic performance evolves over time following the occurrence of faults, since fault development in the CSTR process is inherently dynamic. This temporal analysis provides insight into the generalization ability and responsiveness of the proposed AE-based DST framework. Table 11 presents the comparative performance of the baseline autoencoders and their DST-integrated variants in terms of false alarm rate, geometric mean, and mean detection delay. Incorporating DST consistently improves model reliability across metrics. Reduced FAR prevents spurious alarms, higher G-mean balances sensitivity and specificity, and shorter MDDs enable timely interventions to prevent fault escalation.

**Table 11:** Comparative Performance of Baseline and DST-Enhanced Autoencoder Models in Terms of FAR, G-Mean, and MDD

Models	FAR (%)	G-mean	MDD (mins)
VAE	2.14	0.793	3.20
SAE	1.90	0.814	0.25
DAE	1.53	0.892	0.69
VAE-DST	2.00	0.826	1.33
SAE-DST	1.69	0.874	0.166
DAE-DST	1.41	0.911	0.56

Among the evaluated models, the SAE-DST exhibits outstanding fault detection, achieving an extremely low MDD (0.166 min) alongside minimal FAR (1.69%) and high G-mean (0.874), indicating both rapid response and reliable classification. DAE-DST also improves detection efficiency, reducing MDD from 0.69 to 0.56 min while increasing G-mean to 0.911. VAE-based models show slower detection (MDD 3.20 min), although DST integration still provides modest improvements in reliability (MDD reduced to 1.33 min). Performance of the sparse autoencoder model in terms of FAR, G-mean, and MDD is presented in Table S6. These findings validate DST integration as a robust fusion framework that enhances autoencoder-based fault diagnosis by reducing false alarms, improving balanced accuracy, and shortening detection delays, demonstrating its potential for reliable, responsive process monitoring in complex systems like the CSTR.

#### 4.5.7 Comparison with Representative Fault Diagnosis Methods

Table 12 summarizes reported classification accuracies across representative chemical process benchmarks. Given the differences in process dynamics, data sets, fault configurations, and evaluation procedures, these results are used for reference rather than as direct performance benchmarks. On the CSTR benchmark, DAE-DST achieved the highest accuracy among the evaluated AE-based DST variants (93%), followed by SAE-DST (92%) and VAE-DST (90%). These results indicate that integrating DST with different autoencoder models can effectively reduce classification uncertainty and support stable fault diagnosis. Among the reported methods, CNN-DAE achieved 89% accuracy on a depropanizer distillation process,^71^ DisAE achieved 89% on a penicillin fermentation process,^72^ and RATransformer achieved 91% on the Tennessee Eastman process.^58^ Other reported approaches include CNN (72–90%), VAE (86%), ALSTM-FCN (89%), and KSCW-VAE (91%) across different chemical process benchmarks.

**Table 12:** Reported Classification Performance of the Proposed Framework and Representative Existing Approaches

Methods	Chemical process	Accuracy (%)	Important highlights	Reference
DAE-DST	CSTR process	93	Demonstrates notable performance gains	This study
SAE-DST	CSTR process	92	Strengthens decision reliability	This study
VAE-DST	CSTR process	90	Reduces classification uncertainty	This study
Transformer	Tennessee Eastman process	76	Exhibits better detection performance	^2^
CNN	Tennessee Eastman process	72	Exhibits good detection performance	^2^
CNN-DAE	Depropanizer distillation process	89	Achieves the highest accuracy	^71^
CNN	Reactive distillation for carbonate ester production	90	Shows remarkable performance	^73^
DisAE	Penicillin fermentation process	89	Demonstrates superior classification performance	^72^
VAE	Tennessee Eastman process	86	Performs effectively in detecting faults	^74^
RATransformer	Tennessee Eastman process	91	Achieves the highest average accuracy	^58^
SDAE	CSTR process	86	Exhibits superior classification performance	^58^
ALSTM-FCN	Tennessee Eastman process	89	Achieves improved performance	^75^
KSCW-VAE	Metallurgical roasting process	91	Delivers superior classification accuracy	^76^

The consistent performance of the proposed framework on the CSTR benchmark demonstrates the applicability of evidence-based fusion to nonlinear process fault diagnosis, while the literature results provide a reference for existing approaches evaluated under different process settings.

## 5 Conclusion and Future Work

This work presents a novel framework for intelligent process monitoring in nonlinear chemical processes, successfully integrating autoencoder-based models with Dempster–Shafer theory (DST) to enhance diagnostic reliability under uncertainty. The framework was developed to address key challenges in chemical-process fault diagnosis, including data scarcity, measurement uncertainty, computational efficiency, and detection latency. Sparse and variational autoencoders addressed data scarcity by learning compact latent representations, while probabilistic feature encoding and DST fusion reconciled conflicting diagnostic evidence to reduce measurement uncertainty. Compact feature extraction and rapid inference (0.0026 ms/sample), combined with DST-based decision fusion, improved detection performance and fault isolation, with uncertainty quantification improving overall diagnostic reliability.

Validation on the nonlinear CSTR benchmark demonstrated that AE-based DST integration improved the diagnostic performance of the standalone autoencoders. Baseline DAE, SAE, and VAE models achieved 87%, 79%, and 74% accuracy, respectively, whereas DST integration improved performance substantially: DAE–DST reached 93% accuracy, with balanced precision and F_1_-scores of 90%, a G-mean of 0.911, a false alarm rate of 1.41%, and an MDD of 0.56 min; SAE–DST achieved 92% accuracy with the fastest MDD of 0.166 min; and VAE–DST improved to 90% accuracy. Comparisons with CNN–DAE (89%), DisAE (89%), and RA–Transformer (91%) further indicate the effectiveness of uncertainty-aware decision fusion under the evaluated benchmark conditions. These results demonstrate the methodological effectiveness of the proposed AE–based DST framework in improving diagnostic accuracy, detection responsiveness, and robustness, supporting reliable real-time deployment in safety-critical chemical processes.

Future research will integrate semisupervised learning and generative augmentation with the proposed framework to exploit unlabeled data and generate synthetic samples for underrepresented faults, thereby improving data efficiency and class balance while preserving uncertainty-aware DST fusion. Further extensions will focus on adaptive learning under dynamic process conditions, explainable decision-making to improve operator trust and practical applicability, and validation on higher-dimensional industrial processes to assess scalability across large sensor networks. Application to other complex chemical processes, including the Tennessee Eastman process, oxygen capture systems, and crude distillation units, will also be investigated to further assess the scalability and generalizability of the proposed framework.

## Supplementary Material



## Data Availability

Data and code will be made available on request.

## Notes

Definitions of basic notations in Dempster–Shafer theory and autoencoder-based models; confusion matrices of the autoencoder-based models for CSTR process monitoring; class-wise ROC analyses of SAE- and VAE-derived latent features; basic probability assignments, belief, and plausibility values for the 12 fault conditions; the derivation of the BPA formulation (PDF)
