# Supplementary material for: Autoencoder-Based Dempster-Shafer Decision-Making Framework for Intelligent Process Monitoring: Applications to Nonlinear Chemical Processes
Source: ACS Omega. 2026 Oct 23;11(43):67522–41. doi: 10.1021/acsomega.6c08762 (PMC13736973; doi:10.1021/acsomega.6c08762)
Supplement: Supplementary file 1 [file ao6c08762_si_001.pdf]

Supplementary Material

Autoencoder-Based Dempster-Shafer Decision-Making Framework for Intelligent Process Monitoring: Applications to Nonlinear Chemical Processes

Remigius Nnadozie Ewuzie [a], Shivaneswar Gunasekaran [a], Zainal Ahmad [a],

Norazwan Md Nor [a,*]

[a] School of Chemical Engineering, Universiti Sains Malaysia, Engineering Campus, Seri Ampangan, 14300 Nibong Tebal, Penang, MALAYSIA.

Tel: +604-5995750

*Email: chnorazwan@usm.my (corresponding author)

Table S1. **Definitions of Basic Notations in Dempster–Shafer Theory**

Notation	Name	Definition of notations
$\Theta = \{F_1, F_2, \ldots, F_{12}\}$	frame of discernment	The 12 mutually exclusive and exhaustive fault hypotheses in the CSTR process.
$F \subseteq \emptyset$	Hypothesis (focal element)	A subset of possible faults.
$m(A)$	Basic probability assignment (BPA) (or mass function)	The belief mass assigned to hypothesis A.
$m(\{F_i\})$	Singleton BPA	Belief mass assigned only to a single fault F_i.
$\sum_{A \subseteq \Theta} m(A) = 1$	Normalization rule	The total mass over all subsets of Θ equals 1.
$m(\emptyset) = 0$	Empty set constraint	No mass is assigned to the empty set.

Table S2. Definitions of Basic Notations in Autoencoder-Based Models

Notation	Name	Definition of notations
x	Input data	Original process measurement fed into the autoencoder.
$\tilde{x}$	Corrupted input	Noisy version of the input used in DAE training.
x_i	Reconstructed output	Output reconstructed by the decoder.
W_{E}, b_{E}	Encoder weights and biases	Parameters used to map input to the latent representation.
W_{D}, b_{D}	Decoder weights and biases	Parameters used to reconstruct the input from the latent space.
h	Latent representation	Encoded feature vector in the hidden layer.
L_{DAE}	DAE loss function	Reconstruction loss between input and output, encouraging noise removal.
p	Sparsity parameter	Desired level of activation for hidden neurons in SAE.
$\hat{\mathrm{p}}_{\mathrm{j}}$	Average activation	Mean activation of hidden neuron j over training samples.
H	Number of hidden neurons	Total neurons in the hidden layer.
$\mathrm{KL}(\mathrm{p}\|\hat{\mathrm{p}})$	KL divergence	Penalty measuring the deviation between the desired and actual sparsity.
L_{SAE}	SAE loss function	A combination of reconstruction loss and sparsity penalty.
z	Latent variable	Hidden representation capturing underlying features.
$\log \mathrm{p}_{\theta}\ (\mathrm{x})$	Marginal log-likelihood	Log probability of observing data under the model.
$\mathrm{L}_{\mathrm{VAE}}$	Evidence lower bound	Optimized objective function balancing reconstruction accuracy and regularization.
θ	Decoder parameters	Trainable weights of the decoder network.
ϕ	Encoder parameters	Trainable weights of the encoder network.

Table S3. **The Measured Variables in the CSTR Process**

Number	Process variable
1	Concentration of A in the inlet flow
2	Temperature of the inlet flow
3	Temperature of the inlet coolant flow
4	Coolant flow-rate
5	Concentration of B in the outlet flow
6	Temperature of the outlet flow
7	Temperature of the outlet coolant flow.

Table S4. Classification of CSTR process faults by fault profile.

Fault number	Description of faults	Fault type	Fault profile
Fault 1	Catalyst decay	Process Fault	Slow drift
Fault 2	Heat transfer fouling	Process Fault	Slow drift
Fault 3	Bias in the input concentration sensor	Sensor Fault	Step
Fault 4	Bias in the inlet temperature sensor	Sensor Fault	Step
Fault 5	Bias in the coolant inlet temperature sensor	Sensor Fault	Step
Fault 6	Bias in the output concentration sensor	Sensor Fault	Step
Fault 7	Bias in the reactor temperature sensor	Sensor Fault	Step
Fault 8	Bias in the coolant temperature sensor	Sensor Fault	Step
Fault 9	Bias in the coolant flow rate sensor	Sensor Fault	Step
Fault 10	Disturbance in feed concentration	Process Disturbance	Random variation in feed concentration
Fault 11	Disturbance in the reactant feed temperature	Process Disturbance	Random variation in reactant feed temperature
Fault 12	Disturbance in coolant feed temperature	Process Disturbance	Random variation in coolant feed temperature

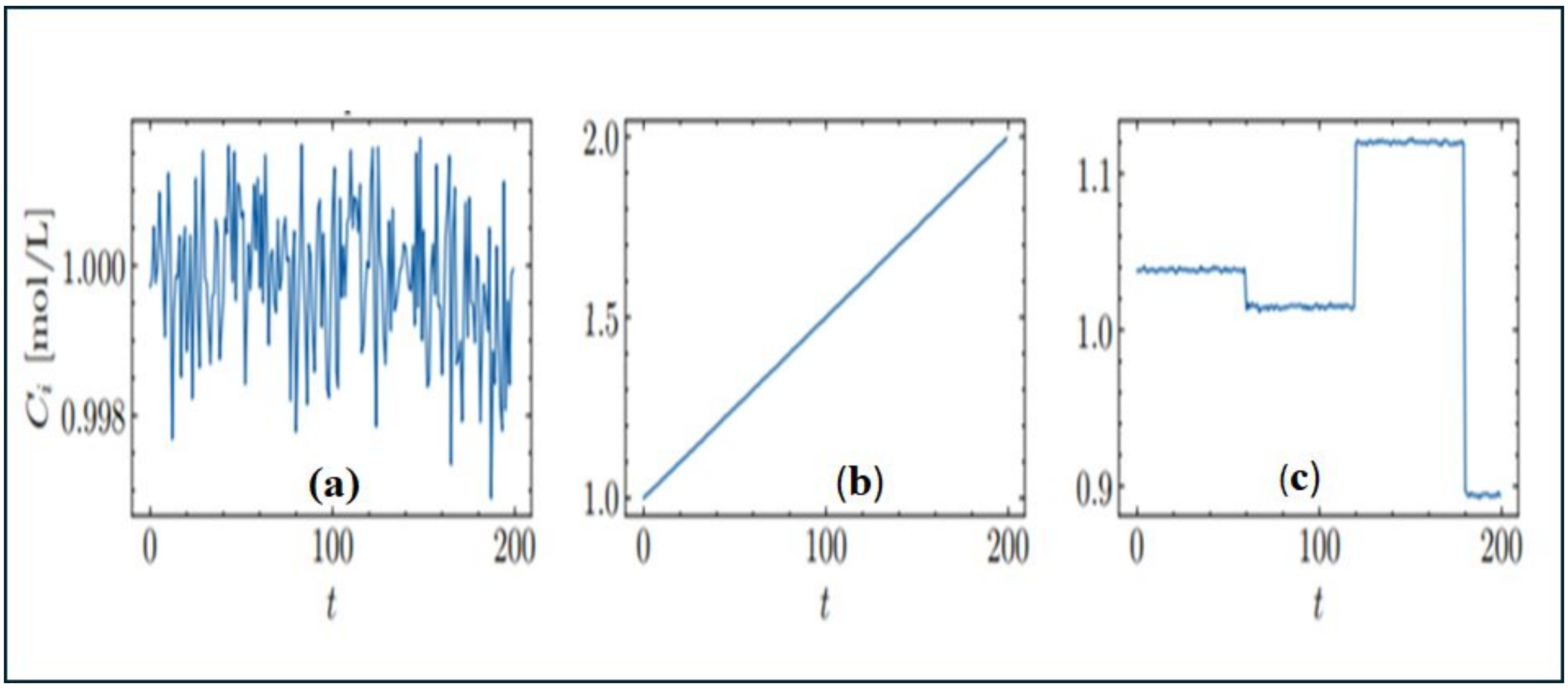

Figure S1. The behavior of the concentration variable (C_i) under: (a) normal operation condition, (b) sensor bias, (c) process disturbance.

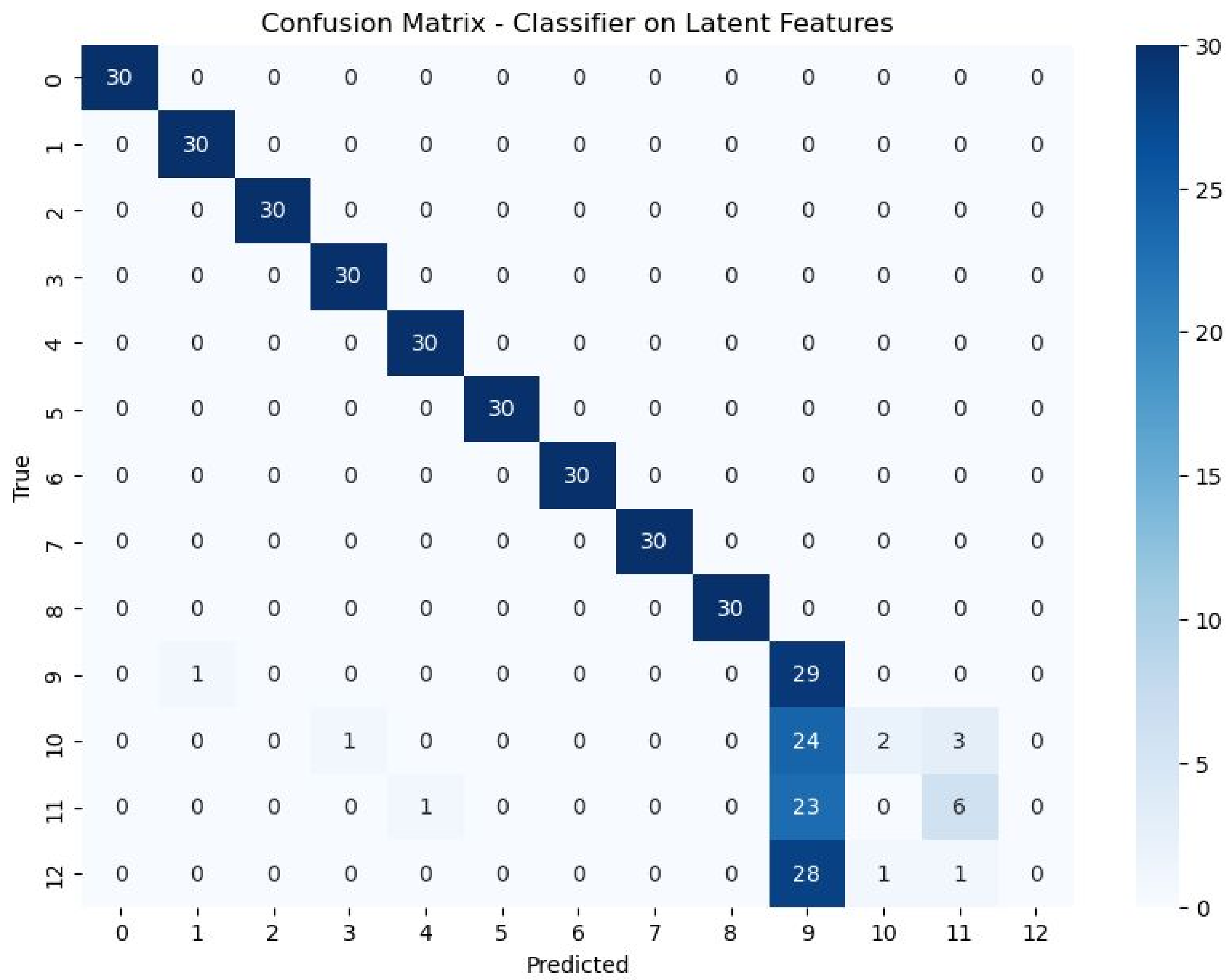

Figure S2. Confusion matrix of the sparse autoencoder for CSTR process monitoring.

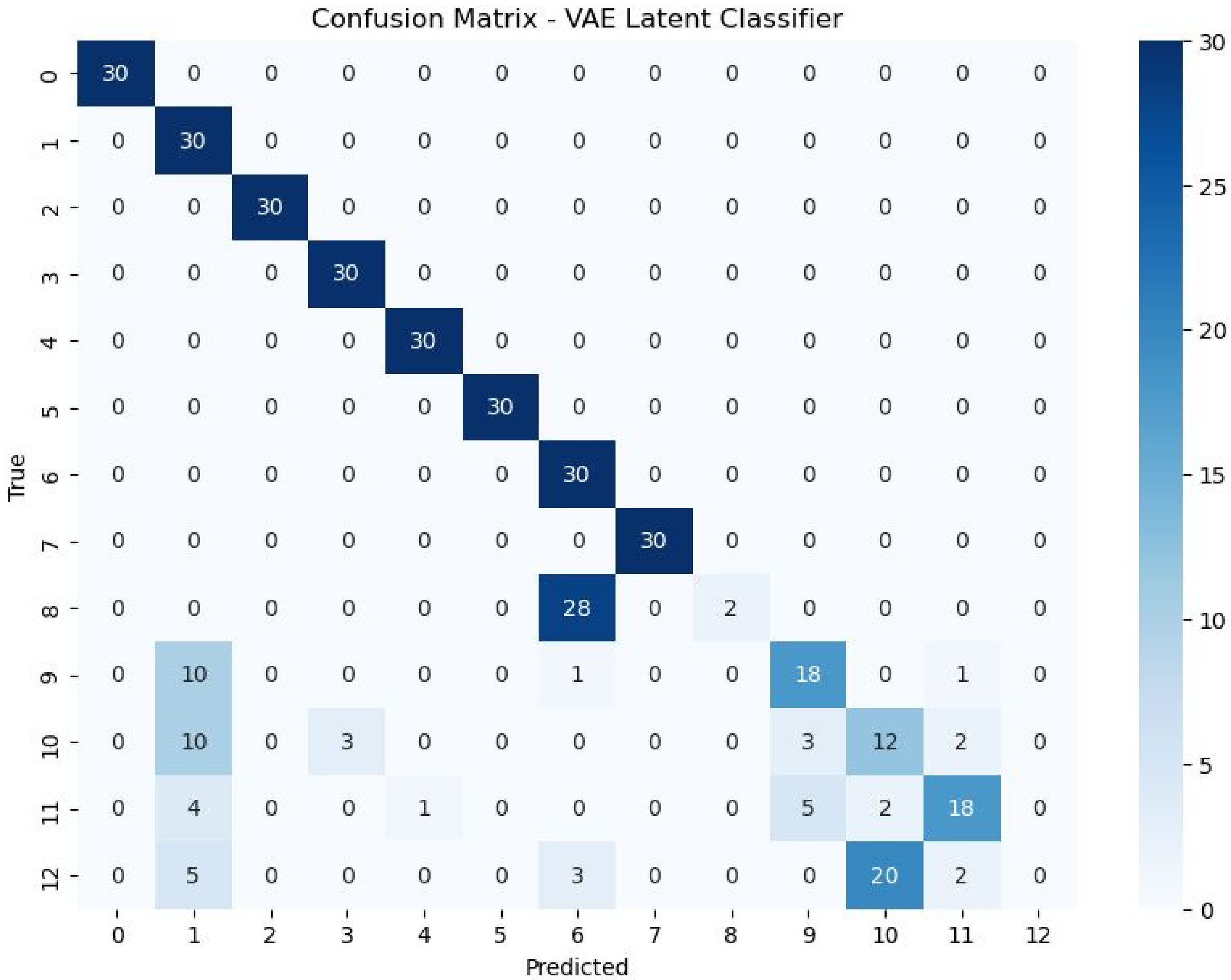

Figure S3. Confusion matrix of the variational autoencoder for CSTR process monitoring.

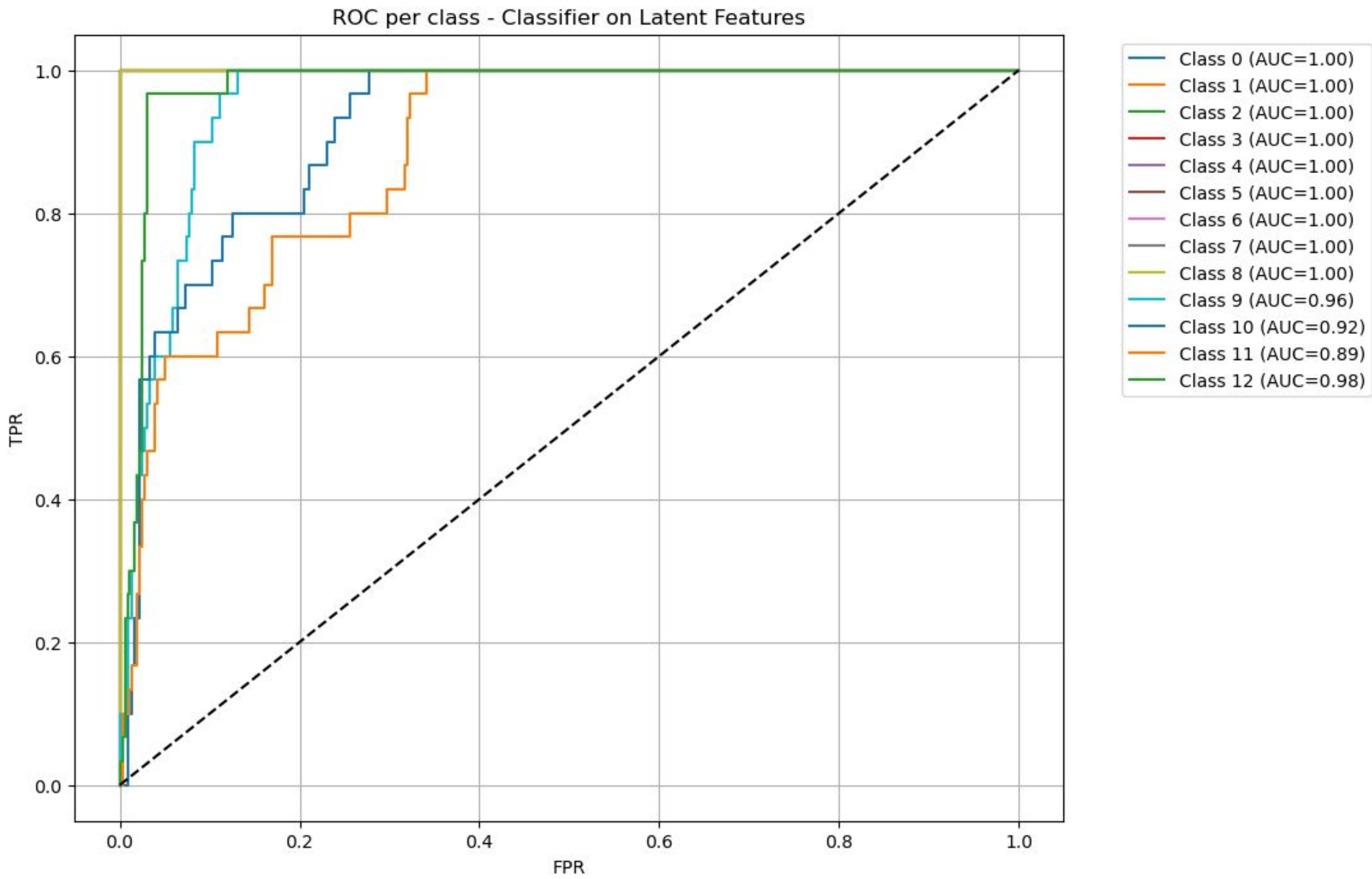

Figure S4. Class-wise ROC analysis of SAE-derived latent features for CSTR process monitoring.

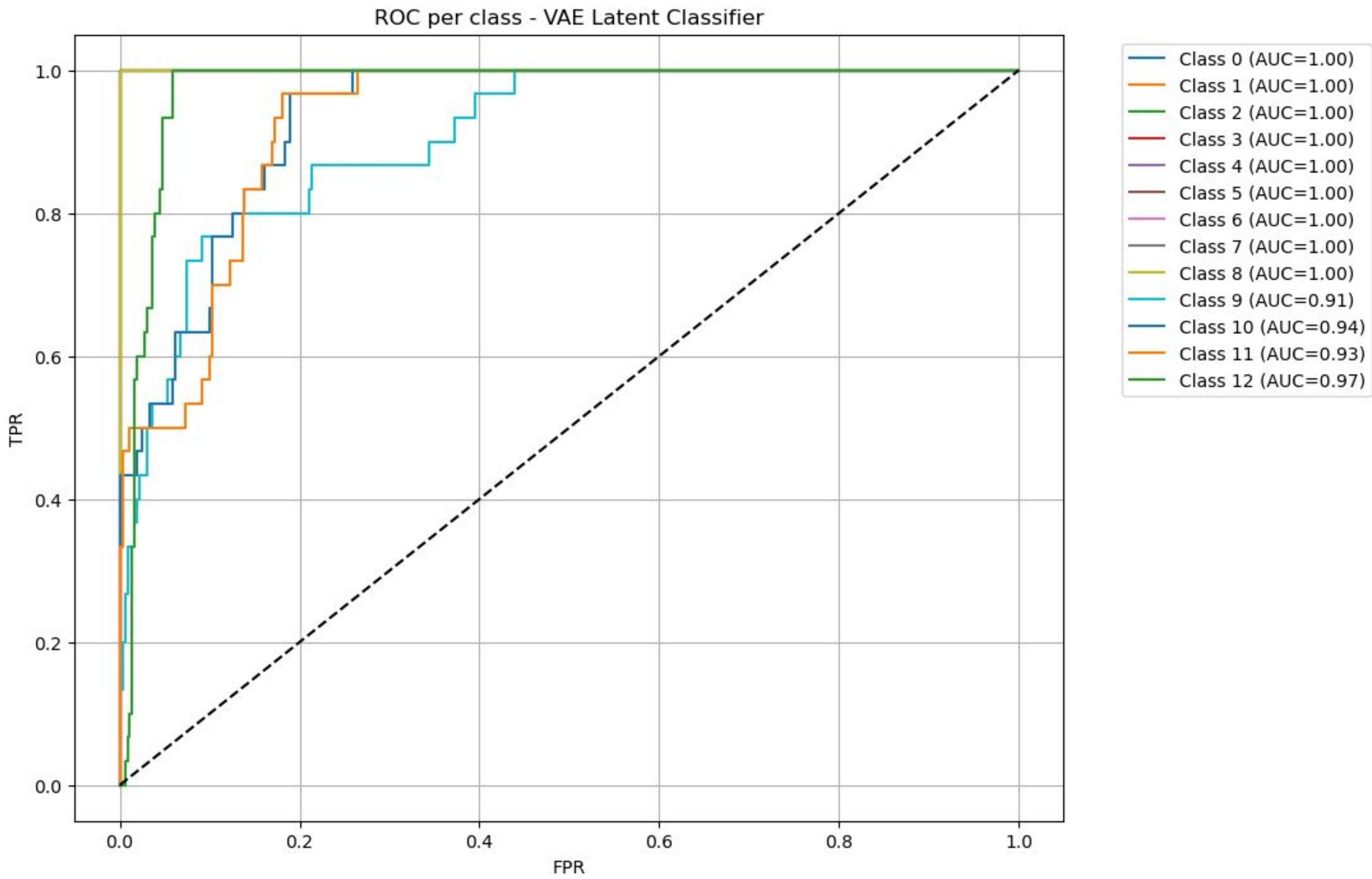

Figure S5. Class-wise ROC analysis of VAE-derived latent features for CSTR process monitoring.

Table S5. Basic Probability Assignments, Belief, and Plausibility Values for 12 Fault Conditions

Fault number	BPA $m(\{F_i\})$	Belief $\mathrm{Bel}(\{F_i\})$	Plausibility $Pl(\{F_i\})$
Fault 1	0.0970	0.0970	0.0970
Fault 2	0.0970	0.0970	0.0970
Fault 3	0.0970	0.0970	0.0970
Fault 4	0.0970	0.0970	0.0970
Fault 5	0.0970	0.0970	0.0970
Fault 6	0.0970	0.0970	0.0970
Fault 7	0.0970	0.0970	0.0970
Fault 8	0.0970	0.0970	0.0970
Fault 9	0.0553	0.0553	0.1053
Fault 10	0.0747	0.0747	0.2047
Fault 11	0.0553	0.0553	0.1153
Fault 12	0.0388	0.0388	0.1788

The BPA formulation is given in **the equations. (26**) and (**27**) of the main manuscript are elaborated here in a step-by-step manner.

ACC_i denotes the classification accuracy of the fault class F_i where $i = 1, 2,3,....n$ and n is the total number of fault classes.

Accuracy corresponding to the fault class F_i:

$$ACC_1,\ ACC_2,\ ACC_3,........12 = 100\%, 100\%, 100\%,....40\% \qquad S1$$

Convert percentages into decimal values:

$$ACC_1,\ ACC_2,\ ACC_3,........12 = 1.00, 1.00, 1.00,....0.40 \qquad S2$$

The total sum of classification accuracies:

$$\sum_{i=1}^{n} Acc_i = 1.00 + 1.00 + 1.00 + \ ..... + 0.40 = 10.31 \qquad S3$$

The basic probability assignments for each fault class are expressed as:

$$m(\{F_i\}) = w_{1,}\ w_2\ w_3,....n = \frac{ACC_1}{\sum ACC_i}, \frac{ACC_2}{\sum ACC_i}, \frac{ACC_3}{\sum ACC_i},....n \qquad S4$$

$$m(\{F_i\}) = \frac{1.00}{10.31}, \frac{1.00}{10.31}, \frac{1.00}{10.31}, ...\frac{0.40}{10.31} \qquad S5$$

$$m = 0.0967, 0.0967, 0.0967,....0.0388 \qquad S6$$

where, $m(\{F_i\})$ denotes the reliability-based mass assigned to fault hypothesis F_i *and* w_i represents the normalized reliability weight derived from the corresponding fault-specific classification accuracy.

The weighted sum of the individual accuracies:

$$F_i = \sum_{i=1}^{n} w_i.ACC_i \qquad S7$$

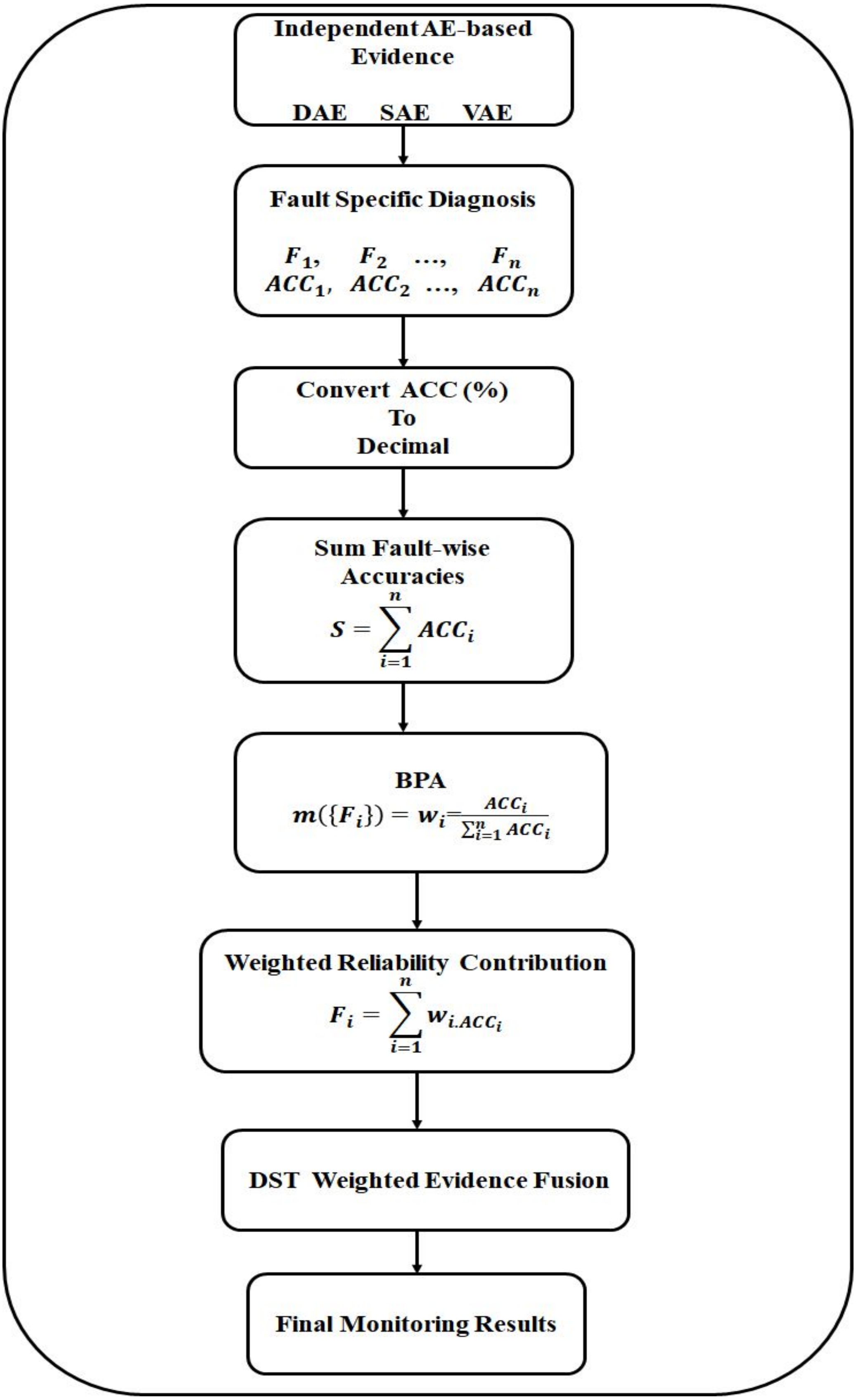

Figure S6. Mathematical workflow for BPA generation and DST-based weighted evidence fusion.

Table S6. Performance of the Sparse Autoencoder Model in Terms of FAR, G-mean, and MDD

Fault number	FAR (%)	G-mean	MDD (mins)
Fault 1	0.0167	0.992	0.00
Fault 2	0.0000	1.000	0.00
Fault 3	0.0000	1.000	0.0
Fault 4	0.0028	0.999	0.00
Fault 5	0.0250	0.987	0.00
Fault 6	0.0083	0.999	0.00
Fault 7	0.0278	0.999	0.00
Fault 8	0.0028	0.999	0.00
Fault 9	0.1528	0.713	1.0
Fault 10	0.0028	0.547	0.00
Fault 11	0.0028	0.365	2.00
Fault 12	0.0000	0.000	0.00
